# Multiple adhesion molecules act together in oligodendrocyte-mediated axonal selection and myelin formation

**DOI:** 10.1371/journal.pbio.3003854

**Published:** 2026-06-25

**Authors:** Swathi Radha, Martina Arends, Georg Kislinger, Agata Rhomberg, Martina Schifferer, Minou Djannatian, Mikael Simons

**Affiliations:** 1 Institute of Neuronal Cell Biology, Technical University of Munich, Munich, Germany; 2 German Center for Neurodegenerative Diseases, Munich, Germany; 3 Munich Cluster of Systems Neurology, Munich, Germany; 4 Department of Neurology, School of Medicine and Health, Technical University of Munich, Munich, Germany; 5 Institute for Stroke and Dementia Research, University Hospital of Munich, LMU Munich, Munich, Germany; Oregon Health and Science University, UNITED STATES OF AMERICA

## Abstract

Rapid information processing in complex organisms depends on myelin, which consists of a multilamellar membrane that tightly adheres to the axonal surface along the internode and at paranodal loops, where specialized adhesion proteins maintain axon-glial contact. Because the decision to myelinate an axon profoundly influences neuronal transmission, this process must be precisely regulated. Yet, it remains unclear which specific molecules enable oligodendrocytes to select appropriate axonal substrates for myelination. Several key myelin-associated adhesion systems have been identified, including Myelin-associated glycoprotein (Mag) and Cell Adhesion Molecule 4 (Cadm4) at the internode, as well as Contactin1 (Cntn1) at the paranode; however, these three adhesion molecules have not previously been deleted in combination. Here, using zebrafish, we systematically disrupted all three myelin-associated adhesion systems. We found that the combined loss of Mag, Cadm4, and Cntn1 severely impairs myelin initiation and destabilizes the few nascent sheaths that do form, resulting in a phenotype characterized by oligodendrocytes exhibiting membrane “stubs”. The failure to form myelin triggered cell death of early myelinating oligodendrocytes and resulted in profound hypomyelination. Our findings reveal that axonal target selection and myelin formation depend on a redundant set of adhesion molecules, and that their simultaneous loss largely abolishes myelin biogenesis.

## Abstract 2

Rapid information processing in the vertebrate nervous system depends on myelin, but how CNS axons are selected for myelination is not well understood. This study in zebrafish shows that axonal target selection by oligodendrocytes depends on a redundant set of adhesion molecules, and that their simultaneous loss largely abolishes myelin biogenesis.

## Introduction

Nervous system function critically depends on myelin, which insulates axons and restricts action potentials to nodes of Ranvier, enabling efficient saltatory conduction [1,2]. The decision to myelinate an axon occurs across multiple stages, from early axon selection to myelin sheath initiation and growth, and ultimately to the refinement and long-term stabilization of sheaths [1,2]. Oligodendrocytes (OL) possess a striking intrinsic ability to initiate myelination, even in culture, where they form myelin around inert plastic fibers above a ~0.4 μm diameter threshold [3–5]. Although axonal diameter influences myelination probability, and axons larger than 1 µm are generally myelinated in the CNS [6], we still do not understand on which basis axons <1 μm are selected for myelination. Because myelination shapes neuronal circuits by optimizing conduction velocity [7], it is of fundamental importance to identify such axon selection criteria.

One such criterion, which has been heavily studied in the past decade, is neuronal activity, linking sensory input and experience-dependent plasticity to myelination [7–10]. In zebrafish, non-synaptic vesicle release in specific axon subtypes occurs at future myelination sites and regulates myelin sheath number [11–14]. However, blocking neuronal activity does not prevent myelin initiation and reduces overall myelin coverage by only ~40%, suggesting that activity is not required for the initial decision to myelinate [13]. Instead, additional repulsive or instructive axonal cues are thought to be critical for axonal targeting and myelin formation [15]. OL processes dynamically contact axons during target selection, where they likely sense molecular cues decisive for the initiation of the myelination program [16,17]. Several molecules that promote axon-OPC interactions and myelination in the CNS have been identified, including axonal EphA4/B1 receptors, integrins, L1-CAM, and N-Cadherin [18–20]. Yet none of these molecules is sufficient on its own to drive the process, leaving the identity of instructive signals unclear. Likewise, neuregulin 1 type III, which is both required and sufficient to induce myelination in the peripheral nervous system (PNS), is largely dispensable for CNS myelination and has only been implicated in activity-dependent myelination [21–23]. In contrast, repulsive molecules such as PSA-NCAM, which delays myelination onset, and JAM2, which prevents ectopic myelination of somas or dendrites, define non-permissive substrates [24,25].

Adhesion molecules are strong candidates for specifying axon-glial contacts and promoting myelination because they physically tether cells, compartmentalize axonal domains, and provide signaling platforms. On the myelinated axon, adhesion molecules are compartmentalized in structural subdomains, mainly the internode (which encompasses the region under compact myelin) and the paranodes (where the cytoplasma-rich edges of the individual myelin lamellae tightly adhere to the axon) that flank the axonal nodes of Ranvier. Previous studies demonstrated that the combined loss of internodal Myelin-associated glycoprotein (MAG) and either of the paranodal proteins Contactin-1 (Cntn1), Contactin-associated protein (Caspr), or Neurofascin B impairs myelin sheath extension and results in aberrant wrapping and the overgrowth of neighboring sheaths [26–28]. Similarly, loss-of-function of Cell Adhesion Molecule 4 (Cadm4), either alone or in combination with internodal MAG or paranodal Caspr, disrupts axo-glial adhesive contacts, impairs myelin growth, and leads to an abnormal organization of axonal domains [29,30]. These studies documented that internodal and paranodal adhesion molecules together play crucial roles in regulating specific aspects of myelin ultrastructure and longitudinal sheath growth. Notably, oligodendrocytes lacking one or even two of these adhesion systems retain the ability to form myelin [26,30–33], raising the possibility that remaining adhesion mechanisms compensate for their loss. Here, we tested the hypothesis that initial axon selection for myelination is governed by a highly redundant set of axonal instructive cues. Using zebrafish, we systematically disrupted three key myelin-associated adhesion molecules: the internodal proteins Mag and Cadm4, and the paranodal protein Cntn1b. We found that the combined loss of these molecules severely impairs both the initiation and stabilization of myelin sheaths, and that axons with diameters <1 μm are significantly less likely to be selected for myelination.

## Results

### Glial Cadm4 and axonal Cadm3 localize to myelin internodes in zebrafish

**Fig 1:**
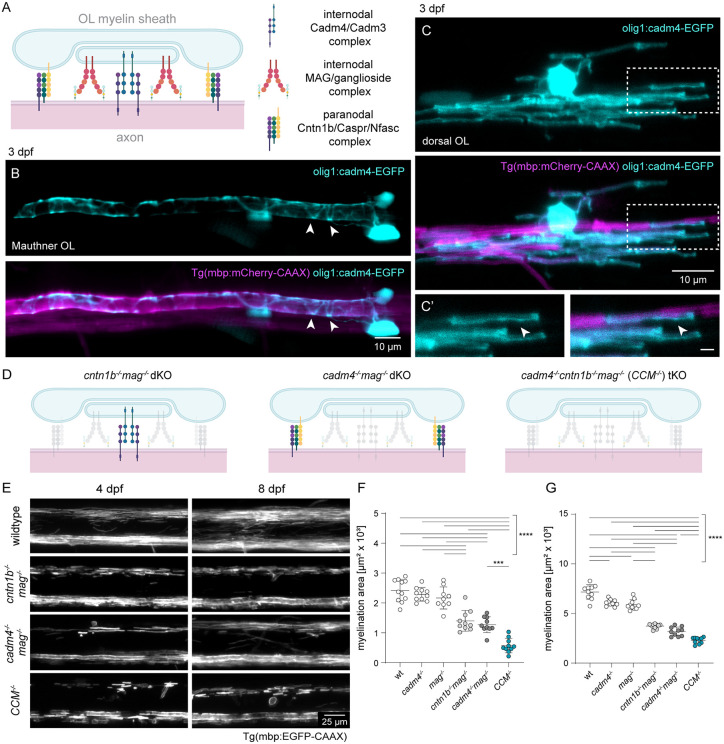
Combined loss of Cadm4, Cntn1b, and Mag leads to severe hypomyelination in the zebrafish spinal cord. **(A)** Schematic showing the localization of axo-glial internodal and paranodal adhesion complexes. **(B)** Confocal image shows a myelinating OL expressing the glial Cadm4 fusion protein olig1:cadm4-EGFP. Cadm4-EGFP (cyan) expression shows a spiraling pattern (arrowheads) across the Mauthner axon, which indicates its localization to the leading edge of the myelin sheath. Myelin sheaths are labeled using the reporter mbp:mCherry-CAAX (magenta). Scalebar is 10 µm. **(C)** Confocal image shows a myelinating OL expressing the glial Cadm4-EGFP (cyan) in a dorsal spinal cord OL at 3 dpf, showing its localization to cytoplasm-rich areas of the myelin sheaths (arrowheads). The OL is co-labeled using the myelin reporter, mbp:mCherry-CAAX (magenta) (**C′** shows zoomed-in images of boxed areas in C). Scale bar is 10 µm for (C) and 5 µm for (C′). **(D)** Schematic showing the loss of axo-glial internodal and paranodal adhesion complexes in the *cntn1b*^*−/−*^*mag*^*−/−*^ dKO, *cadm4*^*−/−*^*mag*^*−/−*^ dKO, and the *CCM*^*−/−*^ tKO. **(E)** Confocal images of myelination in the zebrafish spinal cord in wild type, *cntn1b*^*−/−*^*mag*^*−/−*^ dKO, *cadm4*^*−/−*^*mag*^*−/−*^ dKO, and *CCM*^*−/−*^ tKO at 4 dpf (left panels) and 8 dpf (right panels) using the myelin transgenic reporter line Tg(mbp:EGFP-CAAX). Scale bar is 25 µm. (*CCM*^*−/−*^*: cadm4*^*−/−*^*cntn1b*^*−/−*^ *mag*^*−/−*^). **(F)** Quantification of the area of myelination in the dorsal hemi-spinal cord at 4 dpf. **(G)** Quantification of the area of myelination in the dorsal hemi-spinal cord at 8 dpf. Data was collected from 9-11 fish per genotype for (F and G). Graphs indicate mean values ± SD, analyzed by one-way ANOVA with Tukey’s multiple comparisons test. ***p < 0.001, ****p < 0.0001. Standard deviation projections of the lateral view of the spinal cord shown for (B, C, E). The data underlying this Figure can be found in S1 Data.

Glial Cadm4 (SynCAM4/Necl4) and its main axonal interaction partner, Cadm3 (SynCAM3/Necl1), form a *trans-*interacting complex at the internodal domain of the peripheral axons in mice (Fig 1A) [34–36]. To study Cadm4 localization in zebrafish oligodendrocytes in the CNS, we generated an oligodendrocyte-specific fusion protein, olig1:cadm4-EGFP, and expressed it in the stable transgenic line Tg(mbp:mCherry-CAAX), which labels myelin. Briefly after the onset of spinal cord myelination, at 3 days post fertilization (dpf), Cadm4-EGFP localized to the cytoplasm-rich parts of myelin ensheathing the paired large-diameter Mauthner axons (Fig 1B) and dorsal spinal axons (Fig 1C and 1C′). This mirrored the localization pattern of a Mag fusion protein, olig1:mag-EGFP, which typically localizes to the adaxonal face of the myelin sheath (S1A and S1B Fig) [37]. We then expressed the interaction partner Cadm3 under a pan-neuronal promoter (huC:cadm3-EGFP) in zebrafish carrying a stable transgenic reporter for oligodendrocyte lineage cells and myelin, Tg(sox10:mRFP). Here, Cadm3-EGFP accumulated at the axonal internode at sites of myelin formation, indicating its recruitment to axonal segments engaged in myelin sheath assembly (S1C Fig). In contrast, the axonal fusion protein Caspr-EYFP only initially localized to the internodal domain of the axon (S1D Fig, top), which was followed by a rapid relocation to pre-assembled paranodes during confocal in vivo time-lapse imaging (S1D Fig, middle and bottom, S1 Video), matching the known paranodal localization of Caspr [38–40]. While only 12% of the myelin sheaths exhibited Caspr-EYFP at the paranodes at 2.5 dpf, this proportion increased to 47% by 3 dpf and 91% by 5 dpf (S1E Fig). Together, these expression analyses indicate that Cadm3 and Cadm4 are recruited to the axonal domain during myelination in zebrafish, positioning them to potentially form a *trans-*acting adhesion complex.

### Combined loss of Cadm4, Cntn1b, and Mag adhesion molecules largely abolishes spinal cord myelination

To study how Cadm4 contributes to target recognition during CNS myelination, we generated *cadm4* knockout (KO) zebrafish by CRISPR-Cas9-mediated gene editing. We established stable KOs with an 8-basepair (bp) deletion targeting exon 4, resulting in a premature stop codon immediately downstream of the mutation (S2A–S2E Fig), which leads to a loss of the second Ig-like and the transmembrane domain of Cadm4 (S2B Fig). The successful generation of Cadm4 KO was confirmed by a near-complete loss of *cadm4* mRNA using qPCRs (S2F Fig). Outcrosses with our previously generated *mag*^*−/−*^ and *cntn1b*^*−/−*^*mag*^*−/−*^ knockout lines [26] were done to generate *cadm4*^*−/−*^*mag*^*−/−*^ double knockout (dKO) and *cadm4*^*−/−*^*cntn1b*^*−/−*^*mag*^*−/−*^ triple knockout (tKO, referred to as *CCM*^*−/−*^ tKO throughout the manuscript), respectively. Whereas *cadm4*^*−/−*^*mag*^*−/−*^ dKO fish did not display any abnormal physiological or behavioral patterns, *CCM*^*−/−*^ tKO fish were smaller in size and developed a mild ataxic swimming behavior during adulthood, similar to *cntn1b*^*−/−*^*mag*^*−/−*^ dKO. *CCM*^*−/−*^ tKO had abnormally small egg lays (around 50 eggs per egg lay) with low fertilization rates and a higher mortality rate of young larvae, indicating abnormal fertility and development.

Our analysis in single knockouts, dKOs, and tKOs (Fig 1D) expressing the transgenic myelin reporter Tg(mbp:EGFP-CAAX) revealed a strong reduction of myelin in both the ventral and dorsal spinal cord of *CCM*^*−/−*^ tKOs, with large gaps at 4 dpf that partially persisted by 8 dpf (Fig 1E). Quantification showed that both *cntn1b*^*−/−*^*mag*^*−/−*^ dKO and *cadm4*^*−/−*^*mag*^*−/−*^ dKO fish exhibited a 40%–50% reduction of myelin compared to wild-types, whereas myelin was reduced by over 75% in *CCM*^*−/−*^ tKOs (wt: 2.41 ± 0.37 × 10^3^ µm^2^, *cntn1b*^*−/−*^*mag*^*−/−*^ dKO: 1.39 ± 0.34 × 10^3^ µm^2^, *cadm4*^*−/−*^*mag*^*−/−*^ dKO:1.26 ± 0.26 × 10^3^ µm^2^, and *CCM*^*−/−*^ tKO: 0.57 ± 0.24 × 10^3^ µm^2^, Fig 1F). Myelin content in the *CCM*^*−/−*^ tKOs was persistently reduced by 8 dpf compared to the other genotypes (wt: 7.17 ± 0.74 × 10^3^ µm^2^, *cntn1b*^*−/−*^*mag*^*−/−*^ dKO: 3.73 ± 0.24 × 10^3^ µm^2^, *cadm4*^*−/−*^*mag*^*−/−*^ dKO: 3.18 ± 0.46 × 10^3^ µm^2^, and *CCM*^*−/−*^ tKO: 2.29 ± 0.31 × 10^3^ µm^2^, Fig 1G). Notably, myelin remained patchy along *CCM*^*−/−*^ tKO Mauthner axons at 8 dpf (Fig 1E), which usually start myelinating first in zebrafish and are entirely covered by myelin by 4 dpf [41]. To test how the loss of Cadm4 affected the expression of other Cadm family members, we analyzed the mRNA expression of *cadm1a*, *cadm1b*, *cadm2a*, and *cadm3* in wild-types and *CCM*^*−/−*^ tKOs at 8 dpf. We found a strong reduction of *cadm3* (by 68.9%), the main axonal interaction partner of *cadm4*, but also of *cadm1b* (by 30%) and *cadm2a* (by 40%, S2G Fig). *Cadm2b* was not expressed in either wild-type or *CCM*^*−/−*^ tKO samples at this time point.

### Cadm4, Cntn1b, and Mag are required to initiate myelination and stabilize sheaths

**Fig 2:**
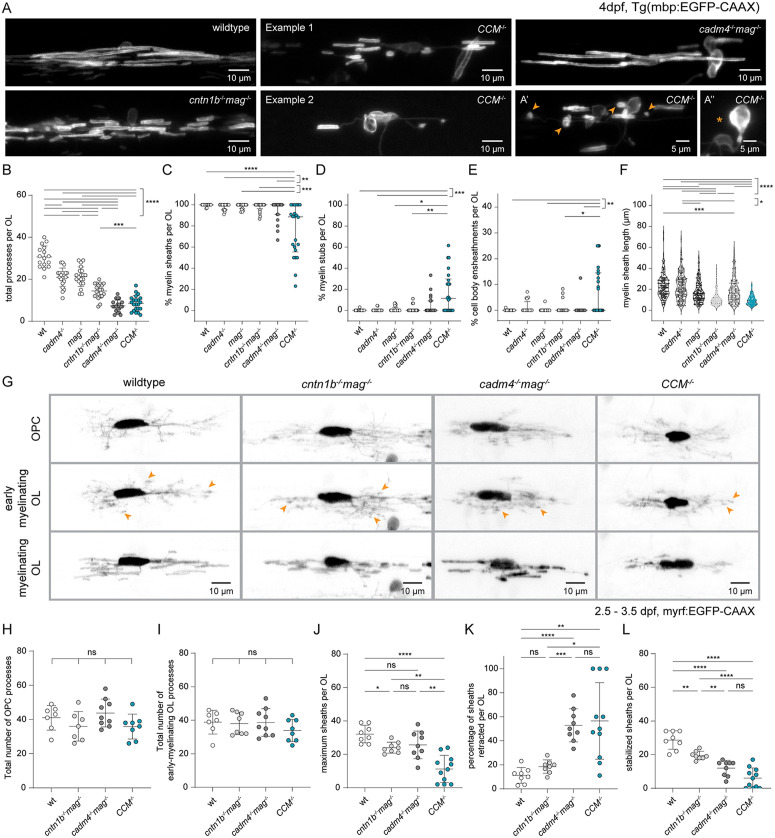
Impaired myelin sheath formation and sheath stabilization in the *CCM*^*−*^^*/*^^*−*^ triple knockout. **(A)** Confocal images of OLs in wild-type, *cntn1b*^*−/−*^*mag*^*−/−*^ dKO, *cadm4*^*−/−*^*mag*^*−/−*^ dKO, and *CCM*^*−/−*^ tKO zebrafish spinal cord at 4 dpf, labeled with the fluorescent reporter Tg(mbp:EGFP-CAAX). Arrowheads indicate membrane stubs **(A′)**, and an asterisk indicates cell body ensheathment **(A″)** in *CCM*^*−/−*^ tKO. Scalebar is 10 µm in (A) and 5 µm in (A′ and A″). **(B–E)** Quantification of the total number of processes per OL in the dorsal spinal cord at 4 dpf (B), and of the percentages of processes connected to myelin sheaths (C), myelin stubs (D), and cell body ensheathments (E). Data was collected from a total of 16 (wt) and 20–22 (rest of the genotypes) individual oligodendrocytes from 9 to 11 fish per genotype. **(F)** Quantification of myelin sheath length in the dorsal spinal cord at 4 dpf. Data was collected from 160 to 180 individual sheaths, and sampled from 3 to 7 fish per genotype. **(G)** Selected frames from confocal time-lapse recording of OL in wild type, *cntn1b*^*−/−*^*mag*^*−/−*^ dKO, *cadm4*^*−/−*^*mag*^*−/−*^ dKO, and *CCM*^*−/−*^ tKO in the zebrafish spinal cord. OLs were sparsely labeled using myrf:EGFP-CAAX and tracked from the OPC stage to the myelinating OLs stage over 13 hours between 2.5 and 3 dpf. Scale bars are 10 µm. Maximum intensity projections shown. **(H)** Quantification of the total number of OPC processes identified at the beginning of the time-lapse imaging. **(I)** Quantification of the total number of early-myelinating OL processes, approximately 2 hours from the OPC stage, when they form nascent myelin sheaths (indicated by arrowheads). **(J)** Quantification of the maximum number of sheaths formed by newly-differentiated OLs during the 13-h time lapse. **(K)** Quantification of the percentage of retracted myelin sheaths of the maximum myelin sheaths formed by individual newly-differentiated OLs during the time-lapse imaging. **(L)** Quantification of the number of stable OL sheaths at the end of the time-lapse imaging. Data was collected from a total of 8–11 individual OLs, and sampled from 4 to 7 fish per genotype for (H–L). Images show standard deviation projections (G). Graphs indicate mean values ± SD (B, H–L), and median with interquartile range (C–F). Data was analyzed by one-way ANOVA with Tukey’s multiple comparisons test (B, H, I), non-parametric Kruskal–Wallis test with Dunn’s multiple comparisons test (C–F), and Brown–Forsythe and Welch ANOVA with Dunnett’s T3 multiple comparisons test (J–L). *p < 0.05, **p < 0.01, ***p < 0.001, ****p < 0.0001. The data underlying this Figure can be found in S1 Data.

To further investigate the observed impairment in myelin initiation, we quantified the total number of processes extended by individual OLs in wild-types and KOs at 4 dpf. While *cadm4*^*−/−*^ and *mag*^*−/−*^ KOs each exhibited approximately a one-third reduction in OL process number compared to wild-types, the combined loss of paranodal and internodal adhesion in *cntn1b*^*−/−*^*mag*^*−/−*^ dKOs led to a greater than 50% reduction in total processes (wt: 30.63 ± 5.25; *cadm4*^*−/−*^: 20.90 ± 4.71, *mag*^*−/−*^: 21.25 ± 4.66, and *cntn1b*^*−/−*^*mag*^*−/−*^ dKO: 14.45 ± 3.47, Fig 2A and 2B). Compared to these KOs, both *cadm4*^*−/−*^*mag*^*−/−*^ dKO and *CCM*^*−/−*^ tKOs were characterized by an even more pronounced, over 75% reduction of total OL processes (*cadm4*^*−/−*^*mag*^*−/−*^ dKO: 7.30 ± 2.79, *CCM*^*−/−*^ tKO: 8.55 ± 3.64, Fig 2A and 2B). Strikingly, over half of the analyzed *CCM*^*−/−*^ tKO OLs, but almost none in the other KOs, exhibited impaired myelin formation. Specifically, this means that only 20%–60% of their processes formed myelin sheaths (defined by a minimum length of >2 µm) (Fig 2C). The remaining processes terminated in stub-like structures (Fig 2D) and some were connected to ectopically ensheathed neuronal cell bodies (Fig 2E). Notably, the few myelin sheaths that did form in *CCM*^*−/−*^ tKOs were significantly shorter than wild-type sheaths, but did not differ in length from *cntn1b*^*−/−*^*mag*^*−/−*^ dKO sheaths (wt: 21.98 [15.52–28.89] µm, *cntn1b*^*−/−*^*mag*^*−/−*^ dKO: 9.030 [6.575–11.87] µm, *cadm4*^*−/−*^*mag*^*−/−*^ dKO: 16.38 [10.15–25.47] µm, and *CCM*^*−/−*^ tKO: 8.920 [5.865–12.23] µm (all values are median [IQR]), Fig 2F).

In order to elucidate how the stub-like membrane structures in *CCM*^*−/−*^ tKO originate, we performed in vivo time-lapse imaging of individual early myelinating OLs over 13 hours starting at 2.5 dpf (Fig 2G–2L and S2–S5 Videos). Oligodendrocyte lineage cells were sparsely labeled using a myrf:EGFP-CAAX transgene, to track OPCs along their differentiation and myelination trajectory as they start to form myelin sheaths (Fig 2G). We found that the OPCs and early-myelinating OLs in the wild types and knockouts formed the same number of processes (Fig 2H and 2I). Newly differentiated wild-type OLs were highly branched and made multiple contacts with axons before initiating myelin sheaths and retracting the excess branches (Fig 2G and S2 Video). The *cntn1b*^*−/−*^*mag*^*−/−*^ dKO OLs initiated sheaths similarly to wild-types but extended their sheaths at a slower pace than wild-types, as previously described ([26], Fig 2G and S3 Video). Strikingly, we found that OLs in the *CCM*^*−/−*^ tKO, but not in the wild types or dKOs, initiated fewer myelin sheaths (wt: 32.13 ± 5.25, *cntn1b*^*−/−*^*mag*^*−/−*^ dKO: 24.0 ± 3.30, *cadm4*^*−/−*^*mag*^*−/−*^ dKO: 25.67 ± 8.26, and *CCM*^*−/−*^ tKO: 11.27 ± 8.10, Fig 2J and S5 Video). Of the initiated sheaths, about 50%–60% were retracted in the *cadm4*^*−/−*^*mag*^*−/−*^ dKO and *CCM*^*−/−*^ tKO (compared to about 10% sheath retraction in the wild type and about 20% in the *cntn1b*^*−/−*^*mag*^*−/−*^ dKO (Fig 2K). Taken together, the number of stabilized sheaths was reduced by about 70% in *CCM*^*−/−*^ tKO compared to wild-types (Fig 2L). The *cadm4*^*−/−*^*mag*^*−/−*^ dKO exhibited about 50% less stabilized sheaths compared to wild type by the end of the acquisitions (at about 3.5 dpf, Fig 2L) and was reduced to the same level as *CCM*^*−/−*^ tKO by 4 dpf (Fig 2B). Taken together, these data show that myelin “stubs” originate from both a failure to initiate myelination and sheath retractions. They further suggest that Cadm4, Cntn1b, and Mag are required together to establish stable axo-glial contacts, form myelin sheaths, and prevent the ectopic ensheathment of neuronal cell bodies.

### Sheath stabilization by axoglial adhesion molecules is required for the survival of early myelinating oligodendrocytes

**Fig 3:**
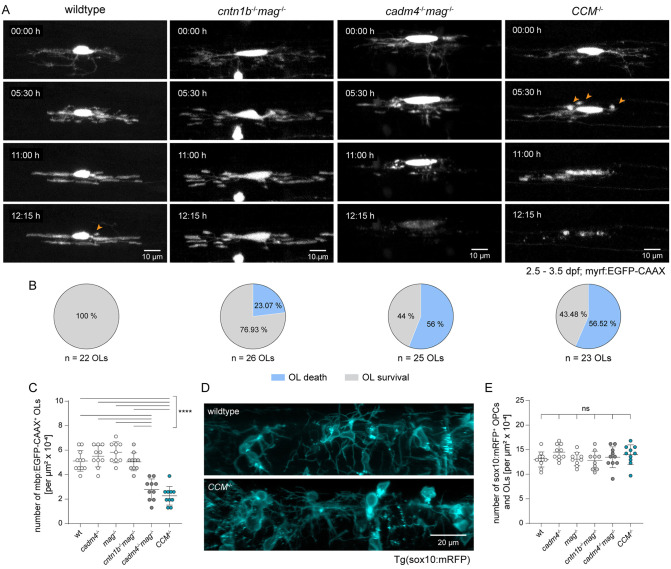
Loss of Cadm4, Cntn1b, and Mag leads to impaired formation and stability of nascent myelin sheaths and cell death of early myelinating OLs. **(A)** Selected frames from confocal time-lapse recording show survival or cell death of individual myrf:EGFP-CAAX-expressing, newly differentiated OLs in wild type, *cntn1b*^*−/−*^*mag*^*−/−*^ dKO, *cadm4*^*−/−*^*mag*^*−/−*^ dKO, and *CCM*^*−/−*^ tKO, imaged over 13 hours between 2.5 and 3.5 dpf. Scalebar is 10 µm. Maximum intensity projections shown. **(B)** Quantification of the percentage of labeled OLs undergoing cell death was analyzed from the time-lapse recording, referred to in (A). Data were collected from 10 fish for wild type, 7 fish for *cntn1b*^*−/−*^*mag*^*−/−*^ dKO, 8 fish for *cadm4*^*−/−*^*mag*^*−/−*^ dKO, and 8 fish for *CCM*^*−/−*^ tKO. **(C)** Quantification of the number of mbp:EGFP-CAAX-expressing myelinating OLs per µm^2^ in the zebrafish dorsal hemi-spinal cord. **(D)** Confocal images of the lateral view of wild-type and *CCM*^*−/−*^ tKO zebrafish dorsal spinal cord at 4 dpf, where OPCs and newly-differentiated oligodendrocytes are labeled with the transgenic reporter line Tg(sox10:mRFP). Images were cropped to exclude myelin-dense regions. Scalebar is 20 µm. **(E)** Quantification of the number of sox10:mRFP-expressing OPCs and newly-differentiated myelinating OLs per µm^2^ in the zebrafish hemi-spinal cord, as identified in (D). Data was collected from a total of 21–26 individual OLs, sampled from 8 to 10 fish (B) and 9–10 fish (C and E) per genotype. Graphs indicate mean values ± SD, analyzed by one-way ANOVA with Tukey’s multiple comparisons test (C and E). ns, non-significant, ****p < 0.0001. The data underlying this Figure can be found in S1 Data.

How does the loss of its myelinating function affect the oligodendrocyte itself? Throughout the time-lapse experiments, we noted that more than half of the *cadm4*^*−/−*^*mag*^*−/−*^ dKO and *CCM*^*−/−*^ tKO OLs suddenly retracted all their processes after initial ensheathment attempts (*cadm4*^*−/−*^*mag*^*−/−*^ dKO: 14 of 25 OLs, *CCM*^*−/−*^ tKO: 13 of 23 OLs). This was followed by fragmentation of the myelin sheaths and cell body, indicating that they were undergoing cell death (Fig 3A and 3B; S8 and S9 Videos). In contrast, none of the wild-type OLs underwent cell death during target selection or myelin initiation (*n* = 22 OLs, Fig 3A and 3B; S6 Video), and *cntn1b*^*−/−*^*mag*^*−/−*^ dKO OLs, of which 23% died at this stage (*n* = 26 OLs, Fig 3A and 3B; S7 Video). When we analyzed the abundance of myelinating OLs across all genotypes at 4 dpf, both *cadm4*^*−/−*^*mag*^*−/−*^ dKOs and *CCM*^*−/−*^ tKOs, but neither of the other KOs, showed a ~50% reduction of mbp:EGFP-CAAX-expressing OLs compared to wild-types (wt: 5.11 ± 0.84 × 10^−4^ per µm^2^, *cntn1b*^*−/−*^*mag*^*−/−*^ dKO: 5.05 ± 0.74 × 10^−4^ per µm^2^, *cadm4*^*−/−*^*mag*^*−/−*^ dKO: 2.78 ± 0.86 × 10^−4^ per µm^2^, and *CCM*^*−/−*^ tKO: 2.26 ± 0.76 × 10^−4^ per µm^2^, Fig 3C). Notably, we did not observe a compensatory increase of sox10:mRFP-expressing oligodendrocyte precursor cells (OPCs) and newly-differentiated OLs in *cadm4*^*−/−*^*mag*^*−/−*^ dKOs or *CCM*^*−/−*^ tKOs (Fig 3D and 3E). Together, these data suggest that the combined loss of Cadm4, Cntn1b, and Mag leads to a reduction in myelinating oligodendrocytes, most likely due to loss of target engagement followed by increased OL cell death.

### Adhesion molecules determine axonal targeting for myelination

**Fig 4:**
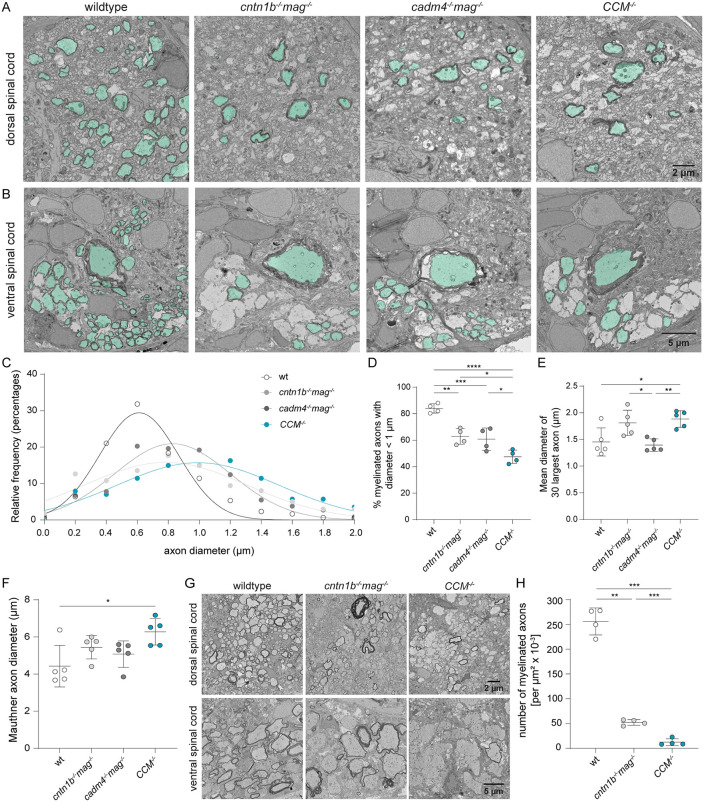
Exacerbated hypomyelination and increased Mauthner axon diameter in the *CCM*^*−*^^*/*^^*−*^ triple knockout. **(A, B)** Scanning electron microscopy (SEM) images of cross-section of wild -type, *cntn1b*^*−/−*^*mag*^*−/−*^ dKO, *cadm4*^*−/−*^*mag*^*−/−*^ dKO, and *CCM*^*−/−*^ tKO zebrafish dorsal (A) and ventral (B) spinal cord at 8 dpf. Pseudo-coloring indicates myelinated axons. Scale bar is 2 µm for (A), 5 µm for (B). **(C)** Quantification of the relative frequency distribution (in percentages) of all the myelinated axons binned by axon diameter. Data points represent means. Lines of best fit with a Gaussian distribution are overlaid. **(D)** Quantification of the percentage of myelinated axons with an axonal diameter <1 µm. **(E)** Quantification of the mean diameter of the 30 largest axons, both myelinated and unmyelinated, in the ventral spinal cord. **(F)** Quantification of the mean Mauthner axon diameter. **(G)** SEM images of the dorsal and ventral wild-type, *cntn1b*^*−/−*^*mag*^*−/−*^ dKO, and *CCM*^*−/−*^ tKO zebrafish spinal cord cross-section at 2.5 months. Scalebar is 2 µm (top panels) and 5 µm (lower panels). **(H)** Quantification of the number of myelinated axons per µm^2^ in wild type, *cntn1b*^*−/−*^*mag*^*−/−*^ dKO, and *CCM*^*−/−*^ tKO at 2.5 months. Graphs represent mean values ± SD, analyzed by one-way ANOVA with Tukey’s multiple comparisons test (D–F), and Brown-Forsythe and Welch ANOVA with Dunnett’s T3 multiple comparisons test (H). *p < 0.05, **p < 0.01, ***p < 0.001, ****p < 0.0001. Data was collected from 4 fish (C, D, H) and 5–6 fish (E and F) per genotype. The data underlying this Figure can be found in S1 Data.

To investigate how the triple loss of adhesion molecules affects oligodendrocyte-mediated axonal target selection and myelin ultrastructure, we performed scanning electron microscopy (SEM) on 8 dpf wild-type, *cntn1b*^*−/−*^*mag*^*−/−*^ and *cadm4*^*−/−*^*mag*^*−/−*^ dKOs, and *CCM*^*−/−*^ tKO spinal cords. We chose this later time point in developmental myelination because it allowed us to capture more myelinated axons. Analysis of ventral and dorsal spinal cord cross-sections revealed a strong reduction of myelinated axons in both dKOs (about 75%–82%) and *CCM*^*−/−*^ tKOs (about 90%) (Figs 4A, 4B, and S3A). Although all sheaths were fully compacted in wild-types at 8 dpf, about 50% of the sheaths in all KOs remained uncompacted at this stage (S3B and S3C Fig), precluding an analysis of myelin thickness by g-ratio quantification. Additionally, we observed ultrastructural myelin aberrations in both *cntn1b*^*−/−*^*mag*^*−/−*^ dKO and *CCM*^*−/−*^ tKOs, in which about 10% of the axons were ensheathed by double or multiple myelin sheaths (“multi-myelin,” S3D and S3E Fig). In contrast, myelin outfoldings, which we previously reported in mutant mice lacking Cntn1 and MAG, were only inconsistently increased in *cntn1b*^*−/−*^*mag*^*−/−*^ dKOs and *CCM*^*−/−*^ tKOs compared to wild-types (S3D and S3F Fig).

To study whether the triple loss of adhesion molecules affects oligodendrocyte-mediated axonal target selection based on their caliber, we further analyzed the distribution of myelinated axons across the whole spinal cord by binned axon diameters. We found that axons below 1 µm were less frequently myelinated in *CCM*^*−/−*^ tKOs, and to some extent also in dKOs (Fig 4C). Compared to wild-types, the percentage of myelinated axons <1 µm was reduced in both dKOs and to a larger extent in *CCM*^*−/−*^ tKO (wt: 84.0 ± 3.48, *cntn1b*^*−/−*^*mag*^*−/−*^ dKO: 62.84 ± 5.97, *cadm4*^*−/−*^*mag*^*−/−*^ dKO: 60.74 ± 8.42, and *CCM*^*−/−*^ tKO: 47.48 ± 4.97, Fig 4D). Because the distribution of myelinated axons showed a shift towards larger-diameter axons in the dKOs and tKO, we tested whether dKOs and tKO actually had increased axon sizes compared to wild-type. In the ventral spinal cord, the 30 largest axons, corresponding to the reticulospinal axons, indeed exhibited significantly larger axon diameters in both *cntn1b*^*−/−*^*mag*^*−/−*^ dKOs and *CCM*^*−/−*^ tKOs (Fig 4E). The Mauthner axon in particular, which is the only axon in the spinal cord that increases its diameter during development, was significantly larger in *CCM*^*−/−*^ tKOs and, to a lesser extent, in dKOs (wt: 4.42 ± 1.11 µm, *cntn1b*^*−/−*^*mag*^*−/−*^ dKO: 5.44 ± 0.62 µm, *cadm4*^*−/−*^*mag*^*−/−*^ dKO: 5.08 ± 0.71 µm, and *CCM*^*−/−*^ tKO: 6.27 ± 0.71 µm, Fig 4F). To test whether myelination defects improve or even resolve later during development, we additionally performed SEM on adult (2.5-month-old) wild-type, *cntn1b*^*−/−*^*mag*^*−/−*^ dKO, and *CCM*^*−/−*^ tKOs zebrafish spinal cords. While many more axons were myelinated in the wild-types, both the *cntn1b*^*−/−*^*mag*^*−/−*^ dKOs and *CCM*^*−/−*^ tKOs continued to exhibit strong hypomyelination, which was much more pronounced in the *CCM*^*−/−*^ tKOs (wt: 256.0 ± 27.21 × 10^−3^ axons per µm^2^, *cntn1b*^*−/−*^*mag*^*−/−*^ dKO: 52.29 ± 5.71 × 10^−3^ axons per µm^2^, and *CCM*^*−/−*^ tKO: 12.36 ± 7.0 × 10^−3^ axons per µm^2^, Fig 4G and 4H). Together, these ultrastructural analyses indicate that Cadm4, Cntn1b, and Mag together are especially required for the selective myelination of axons below 1 µm, and their combined loss alters axonal targeting.

## Discussion

Oligodendrocytes extensively probe their axonal targets by process extension and retraction before they start to make myelin. While they may partially respond to factors like axonal diameter and neuronal activity, a fundamental question remains unsolved: do molecular axonal cues exist that instruct oligodendrocytes to initiate myelination? In this study, we demonstrate that the adhesion molecules Cadm4, Cntn1b, and Mag are key determinants of axoglial contact formation, initiation of the myelination program, and stability of nascent myelin sheaths. Given that these molecules localize to distinct axonal subdomains, how do they act together to instruct myelination?

We found that OPCs in the *CCM*^*−/−*^ tKO extend processes comparable to those in wild type. However, myelinating oligodendrocytes in the *CCM*^*−/−*^ tKO exhibit impaired myelin sheath formation, accompanied by elevated sheath retraction rates. This results in a pronounced deficit in the number of stable sheaths formed per cell and is ultimately associated with increased cell death. The *cadm4*^*−/−*^*mag*^*−/−*^ dKO displays a similar phenotype, including increased sheath retraction and oligodendrocyte loss, but does not show defects in the initiation of myelin sheaths. Together, these findings indicate that the three adhesive molecules collectively are required for stable axon-glia interactions and for the transition from initial axonal contact to productive myelin sheath.

What downstream mechanisms might explain these effects? In Schwann cells, Cadm4 recruits Par3 to the adaxonal membrane, inducing cell polarity and enabling myelination [42,43]. Cadm4 also regulates choline homeostasis and critically influences the phospholipid composition of the myelin membrane in vitro [44]. Notably, Cadm4 overexpression results in more axoglial contacts but fails to form proper sheaths [31], suggesting that Cadm4 levels must be tightly regulated, with an optimal concentration range permitting maximal sheath formation. MAG, in contrast, regulates the tyrosine kinase Fyn, which modulates cytoskeletal dynamics and promotes local translation of MBP mRNA at axon-glia contact sites [45–48]. Furthermore, the reduced myelination capacity could be due to a cellular stress response and impaired proteostasis. Beyond defects in initial contact formation, *CCM*^*−/−*^ tKO OLs also failed to establish stable myelin sheaths. Most nascent sheaths retracted within hours, leaving behind short “stubs”, indicating a critical role for Cadm4, Cntn1b, and Mag not only in initiating but also in stabilizing early myelination events. We therefore hypothesize that Caspr, which initially localizes to the internodal domain and forms a complex with Cntn1b and Neurofascin B, contributes to the stabilization of initial axoglial contacts (Fig 2J). Once the myelination program is initiated, Caspr relocates to the lateral edges in a pre-formed paranodal complex, which possesses strong adhesive interactions due to its cytoskeletal anchoring, whereas Cadm4 and Mag remain at the internode during the wrapping process. This distribution may be necessary to reduce the adhesive strength of the innermost tongue, which continuously moves around the axon and constantly forms and breaks contacts with the axonal surface.

Loss of OPCs by laser ablation in the zebrafish dorsal spinal cord is quickly compensated for by the increased migration and differentiation of nearby OPCs in the zebrafish spinal cord [17]. This OPC replenishment is reflected in this study, in the abundance of sox10:mRFP-expressing OPCs and early-myelinating OLs in the knockouts, which is comparable to the wild type. OL survival is closely linked to myelination [49–52]. In wild-type zebrafish spinal cords, OL death is minimal during early myelination, whereas more than 50% of early-myelinating OLs in the *cadm4*^*−/−*^*mag*^*−/−*^ dKO and *CCM*^*−/−*^ tKO underwent cell death (Fig 3). This may be linked to the reduced sheath stability in *cadm4*^*−/−*^*mag*^*−/−*^ dKO and *CCM*^*−/−*^ tKO. We have previously shown that retracting sheath fragments and the resulting debris accumulate within lysosomes of the OL soma [53]. Consequently, the failure to stabilize sheaths in *CCM*^*−/−*^ tKO may lead to lysosomal overload with lipid-rich myelin debris, inducing cellular stress and promoting cell death. Alternatively, OLs may depend on axon-derived trophic signals [8], such that the loss or reduction of these cues could contribute to the observed cell death. This might, e.g., occur if critical downstream signaling pathways during the transition to the myelinating state are disrupted [54].

The ability of OLs to ensheath inert substrates, including carbon fibers, polymers, or micropillars, demonstrates that physical cues, such as axon caliber, also contribute to myelination [3,5]. Nevertheless, ensheathment of inert surfaces fails to replicate the precise spatial and temporal patterns necessary for functional neural circuits, suggesting the presence of permissive molecular mechanisms that instruct axon selection and myelin initiation [55,56]. In our study, we found that axons with a diameter <1 µm were preferentially affected by the loss of myelination. Since axons <1 µm can be selectively myelinated in the CNS, they might require adhesion molecules as additional cues for decision-making. Some OLs still formed stable sheaths in *CCM*^*−/−*^ tKOs, possibly reflecting axonal heterogeneity. Certain axons may provide alternative molecular cues, larger diameters, or activity-dependent signals favoring myelination [12,57,58]. Indeed, larger axons were preferentially myelinated in the ventral spinal cord of *CCM*^*−/−*^ tKOs, though many remained unmyelinated in adulthood, implying additional regulatory mechanisms.

Notably, a subset of large-diameter axons belonging to reticulospinal neurons exhibited increased caliber in both *cntn1b*^*−/−*^*mag*^*−/−*^ dKO and *CCM*^*−/−*^ tKO. We therefore speculate that the observed increase in axon diameter reflects cytoskeletal rearrangements and/or reduced cortical tension associated with loss of Caspr. Because this phenotype affected only a small subset of axons, axonal enlargement alone cannot account for the loss of small-diameter myelination in *CCM*^*−/−*^ tKO.

In parallel studies in mouse and zebrafish, we previously demonstrated that the functions of adhesion molecules are largely conserved across species [26]. However, the findings presented here differ from the phenotype reported for *Cadm4*^*−/−*^*/Mag*^*−/−*^ mice [30] in two key aspects. First, *cadm4*^*−/−*^*mag*^*−/−*^ dKO zebrafish at 8 days dpf exhibited a > 50% reduction in spinal cord myelin content, whereas *Cadm4*^*−/−*^*/Mag*^*−/−*^ mice showed only a trend toward reduced myelin in the corpus callosum at 3 months of age. Second, whereas Elazar and colleagues [30] reported multi-myelin profiles in approximately 8% of myelinated *Cadm4*^*−/−*^*/Mag*^*−/−*^ axons, we observed comparable structures only rarely, in 1 of 5 fish (S3E Fig). These discrepancies may reflect differences in anatomical region, developmental stage, or species-specific roles of Cadm4.

In summary, Cadm4, Cntn1b, and Mag constitute a combinatorial adhesion code that regulates myelin initiation and the formation of stable sheaths, axonal targeting, and OL survival. These findings reveal that multiple, redundant adhesion signals are essential for precise CNS myelination. Future studies should evaluate their instructive roles across different CNS regions and species, with potential implications for understanding neurodevelopmental disorders and developing regenerative strategies for diseases such as multiple sclerosis.

## Materials and methods

### Ethics statement

The generation and care of all zebrafish lines, and all the experimental procedures in this study were performed according to the regulations of the local governing body (Sachgebiet 54 – Tierschutz, Regierung von Oberbayern, Germany, Az. 55.2-2532.Vet_02-21-172).

### Zebrafish husbandry

The *cadm4* single knockout (ZFIN: # ZDB-ALT-251203–19), *cadm4;mag* double knockout, and *cadm4; cntn1b; mag (CCM)* triple knockout were generated using CRISPR-Cas9-mediated gene editing and/or by outcrossing with the previously generated double mutants of Tg(*cntn1b*^*+/*−^*mag*^*−/−*^; mbp:EGFP-CAAX, ZFIN: ZDB-FISH-210311-21) and Tg(*cntn1b*^*+/*−^; sox10:mRFP, ZFIN: ZDB-FISH-210311-19). Zebrafish lines were raised at 28.5 °C and a 12-hour day–night cycle at the fish facility of the German Center for Neurodegenerative Diseases (DZNE) in Munich according to the local animal welfare regulations. Eggs were obtained by natural spawning and raised in E3 medium at 28.5 °C. For all experiments in this study, larvae between 2.5 - 8 dpf, and 2.5 months of age were used. From 5 dpf on, larvae were raised in rotifer polyculture medium for 8 dpf imaging or further husbandry to adulthood. All fish were used for experiments at a developmental stage before sex specification occurs.

### Generation of cadm4^−/−^mag^−/−^ and CCM^−/−^ zebrafish

The CRISPR-Cas9 gRNA for *cadm4* was designed based on *in silico* predictions in inDelphi [59], where a gRNA with the target sequence 5′-CTTTCCTCTCCACGGGGATA-3′ was predicted to create a single 8-bp deletion at high frequency (47%), with an 80% probability of a frameshift mutation. The predicted efficiency of gRNA and off-targets was confirmed using CHOPCHOP [60] and CRISPOR [61]. The 20-bp target sequence was synthesized as Alt-R CRISPR-Cas9 crRNA and annealed with the tracrRNA (#1072532) (both from IDT) according to the recommended protocol to obtain the crRNA:tracrRNA complex. For CRISPR-Cas9 gene editing, 1 mM of crRNA:tracrRNA complex was combined with 1.25 mg/ml of the Cas9 protein (PNA Bio, #CB03). This gRNA-Cas9 ribonucleoprotein (RNP) complex was injected into one-cell stage fertilized eggs with a stable transgenic background of Tg(mbp:EGFP-CAAX) or Tg(sox10:mRFP). Frame-shift mutations in the outcrossed F1 generation were validated by Sanger sequencing (Eurofins Genomics), and heterozygous fish with identical 8-bp mutations were incrossed to obtain homozygous F2 knockouts.

Genotyping was based on a standard protocol as described in Hruscha and colleagues [62]. Genomic DNA was obtained from F0 larvae or finclips from the F1 adult fish by treating with 1.7 mg/ml Proteinase K (Roche, #39450-01-6) in 1× Tris-EDTA buffer (TE) (pH = 8.0) for 4 hours at 55 °C. PCR primers for knockout validation, forward: 5′-AGGGTTCAAATTTGTGCAGAGT-3′ and reverse: 5′-CGAGCTAATAACAGGCGCTAAT-3′, were chosen from the predicted CHOPCHOP primers. The lysis product was used as PCR template, along with 10 µM of each primer, and amplified with GoTaq G2 DNA Polymerase (Promega, # M7845) using the following thermocycler program: 95 °C for 2 min, 30 cycles of 95 °C for 30 s, 66 °C for 15 s, 72 °C for 22 s; a final extension step of 72 °C for 5 min. The restriction enzyme BciVI(BfuI) was chosen from CRISPOR predictions. The PCR product was digested using BfuI, an isoschizomer of BciVI (ThermoFisher Scientific, # ER1501) with better restriction digestion efficiency, for 1 hour at 37 °C, and analyzed by gel electrophoresis. The primers sequences and restriction digestion enzymes for genes *cntn1b* (AciI, NEB, # R0551S) and *mag* (HindIII-HF, NEB, # R3104S) were obtained from [26].

The primer sequences for the PCR are:

*cadm4*_F: 5′-AGGGTTCAAATTTGTGCAGAGT-3′
*cadm4*_R: 5′-CGAGCTAATAACAGGCGCTAAT-3′
*cntn1b*_F: 5′-CGTCTTTAAATTTTACCTTAAGTGCC-3′
*cntn1b*_R:5′-TGCACTTTAACACAGATTAATGGAA-3′
*mag*_F: 5′-CTCTTTCTCTAAACAGATGCAAGC-3′
*mag*_R: 5′-CGACAGAATTTTCATTGCTGG-3′

### RNA isolation and quantitative PCR

For the analysis of gene expression, 8 dpf zebrafish larvae (wild-type and *CCM*^*−/−*^ tKO) were first anesthetised in tricaine, snap-frozen in liquid nitrogen, and stored at −80°C. RNA isolation was performed using the RNAeasy Mini Kit (Qiagen, #74104) following the manufacturer’s protocol. Frozen single larvae were homogenized in RLT Buffer Plus (Qiagen, #1053393) with β-Mercaptoethanol, using a 200 µl pipette to ensure complete dissociation. The lysate was separated using the QIAshredder spin columns (Qiagen, #79656), followed by purification steps and final reconstitution as per the manufacturer’s protocol. Isolated RNA was reverse transcribed using the SuperScript III First-Strand Synthesis system (ThermoFisher, #18080051), and the obtained cDNA was diluted to be used at a final concentration of 1–10 ng/µl for quantitative PCR (qPCR). qPCR reactions were set up in triplicate using the PowerUp SYBR Green Master Mix (Thermo Fisher Scientific, # A25742), and amplification was done using the Applied Biosystems 7500 Fast Real-Time PCR system. The qPCR primer sequences for *eIF1a* gene were obtained from [26]. Gene expression was analyzed by the ΔΔCt method, where ΔΔCt_wild-type_ = ΔCt_wild-type_ − ΔCt_wild-type mean_, and ΔΔCt_knockout_ = ΔCt_knockout_ − ΔCt_wild-type mean_.

The primers used for qPCR are:

*cadm1a*_F: 5′-ACACGAGCAACGGATCTCTC-3′
*cadm1a*_R: 5′-GGACTGGGTACTGAGCAGC-3′
*cadm1b*_F: 5′-GGTTGTGTTCGCCATGCTTT-3′
*cadm1b*_R: 5′-GCTCCTTTAGCTTCGTGGGT-3′
*cadm2a*_F: 5′-ATCGGTGGAATCGTTGCTGT-3′
*cadm2a*_R: 5′-TCCGCTCCTTTGGCTTCATT-3′
*cadm2b*_F: 5′-TGCAAGCAGGTCAGAAGGAG-3′
*cadm2b*_R: 5′-TGTACGCAGCTCCATCATCC-3′
*cadm3*_R: 5′-AAGATGAGACGGTGGCAGTG-3′
*cadm3*_R: 5′-CGAAGTACAGGGTCTGCTGG-3′
*cadm4*_F: 5′-CCGTGGAGAGGAAAGATAAC-3′
*cadm4*_R: 5′-CACCGTAGGAGCAAAGTGAA-3′
*eIF1a*_F: 5′-AGCAGCAGCTGAGGAGTGAT-3′
*eIF1a*_R: 5′-GTGGTGGACTTTCCGGAGT-3′

### Plasmid generation

To study Cadm4, Cadm3, and MAG localization in myelin, the expression plasmids olig1:*cadm4*-EGFP, olig1:*mag*-EGFP, and huC:*cadm3*-EGFP were assembled by Gateway cloning. First, the coding sequences (CDS) of the *cadm4* (ENSDARG00000040291), *cadm3* (ENSDARG00000057013), and *mag* (ENSDARG00000104023) genes were amplified from 8 dpf wild-type cDNA using Q5 High-Fidelity DNA Polymerase (NEB, # M0491S). The amplified CDS with attB overhangs were then cloned into the pDONR221 vector to create pME plasmids using Gateway BP Clonase II (Thermo Fisher, #11789020). pME plasmids were recombined with p5E-olig1(4.2) [55] (kind gift from Tim Czopka), p3E-EGFP-pA and pDest-Tol2CG2 [63] in a Gateway LR reaction using Gateway LR Clonase II plus (Thermo Fisher Scientific, #12538120) to generate the final expression plasmids. Generation of pTol2-UAS:*caspr*-EYFP was previously described [26]. To label newly differentiated and early myelinating OLs, the expression vector myrf:EGFP-CAAX was assembled using the p5E-myrf (kind gift of Jacob Hines), pME-EGFP-CAAX, p3E-pA, and pDest-Tol2CG2 in a Gateway LR reaction (Thermo Fisher Scientific, #12538120).

The primer sequences used for cloning are:

*cadm3*-cds_F: 5′-GGGGACAAGTTTGTACAAAAAAGCAGGCTgccgccaccATGAGCGGGATGCGCGCGC-3′
*cadm3*-cds_R: 5′-GGGGACCACTTTGTACAAGAAAGCTGGGTcGATGAAGTACTCTTTCTTGTCATCCACCCCGG-3′
*cadm4*-cds_F: 5′-GGGGACAAGTTTGTACAAAAAAGCAGGCTgccgccaccATGATGATGGCATTTTCTTCTG-3′
*cadm4*-cds_R: 5′-GGGGACCACTTTGTACAAGAAAGCTGGGTcCAGTAGGTACTCCTTCTTGCCC-3′
GGGGACAAGTTTGTACAAAAAAGCAGGCTgccaccATGAAGGGCTTAGAGCTGCTGCTTTTGCC-3′
*mag*-cds_R: 5′-GGGGACCACTTTGTACAAGAAAGCTGGGTcTTTAGCTTTAATTTCAGTATAGTCACTGCCGTCACC-3′

### Plasmid transgenesis

For sparse labeling of early myelinating oligodendrocytes in time-lapse imaging and fusion protein localization experiments, fertilized zebrafish eggs at the one-cell stage were microinjected with 25 ng/µl of plasmid DNA and 25 ng/µl of Tol2 transposase mRNA. UAS:caspr-EYFP was co-injected with huC:Gal4 to drive neuronal expression (12.5 ng/µl each). Individual zebrafish larvae were screened at 2.5 dpf to select for optimal expression.

### Confocal imaging

For confocal microscopy, larvae were anesthetised using 600 µM tricaine in E3 medium and mounted laterally in 1%–1.5% UltraPure Low Melting Point Agarose (Invitrogen, #16520100) on a glass-bottom imaging dish with 1–2 ml of E3-tricaine solution. Following the imaging, the larvae were excised from the gel and lysed to confirm the genotypes. A Leica TCS SP8 confocal laser scanning microscope with 8 kHz resonant scanner and automated moving stage in a climate chamber set at 28.5 °C was used. Image acquisition was done using a 1.1 NA 40× water-immersive lens. Immersol (Zeiss, #444969-0000-000) was used for overnight imaging. 488, 514, and 552 nm lasers were used along with hybrid detectors in photon counting mode (4× line accumulation mode) to acquire 8-bit depth images. For the single-time point analysis of myelination at 4 dpf and 8 dpf, tile scans with the following parameters were obtained using the LASX software: 6 × 1 tiles, zoom factor 4.0, pixel size of 0.058 × 0.058 µm, and image size of 72.66 × 72.66 µm, covering a total area of around 72 × 435 µm^2^. The z-step size was set to the system-optimized setting of 0.38 µm, with the pinhole set at 1 AU to image the anterior hemi-spinal cord.

For time-lapse imaging, 2.5–3 dpf zebrafish larvae, microinjected with myrf:EGFP-CAAX, were screened to select for larvae with multiple mosaic labeling of pre-myelinating OLs. The pre-myelinating OLs were characterized based on their morphology, with multiple branched processes that did not begin forming sheath-like structures. The imaging was done on LASX Navigation mode, where multiple regions of interest (ROI) in 2–3 larvae were imaged in the same session. The imaging parameters used: pixel size of 0.162 × 0.162 µm, image size of 83.01 × 83.01 µm, z-step size of 0.5 µm, zoom factor of 3.5, and a pinhole size of 1 AU, and 16× line accumulation. Images were acquired every 15 min for 13 hours.

For experiments on Caspr-EYFP localization, Tg(sox10:mRFP) larvae, microinjected with huC:Gal4 and UAS:caspr-EYFP, were screened for Caspr-EYFP expression. Images were acquired in photon-counting mode with an 8× line accumulation and a pinhole at 0.8 AU. For time-lapse acquisitions, a z-stack was acquired every 30 min for 15 hours on 2–3 dpf larvae. Imaging parameters used: pixel size of 0.047 × 0.047 µm, image size of 48 × 48 µm, and a z-step size of 0.5 µm. For quantifications, z-stacks from multiple ROIs per larvae were acquired at 2.5, 3, and 5 dpf. Imaging parameters used: pixel size of 0.063 × 0.063 nm, with an image size of 58 × 58 µm, with a z-step size of 0.3 µm.

Representative images displayed in the figures show either maximum projection (time-lapse images, Fig 3A) or standard deviation projections of the confocal Z-stack. Representative image for OPCs (Fig 2B) is displayed as a snapshot of the 3D volume in IMARIS 9.9, where the clipping plane tool was used to truncate the 3D volume to display only the OPCs. Lateral views of the spinal cord represent dorsal at the top and anterior to the left. In the figures, stable transgenic lines are indicated as “Tg,” and plasmid transgenesis is indicated without “Tg.”

### Scanning electron microscopy

Zebrafish larvae were anaesthetized in tricaine, and the heads, along with a part of their tails, were removed, followed by transfer to the fixative. The adult fish were terminally anesthetized in tricaine, and the trunk (posterior to the operculum) was dissected to obtain 2–3 mm thick cross-section blocks of the trunks that were immediately transferred to the fixative. Samples were fixed in the fixative (2.5% glutaraldehyde (EMS, #16220), 4% paraformaldehyde (EMS, #15710), 3.5% mannitol (Serva, #69-65-8), 2 mM CaCl_2_ in 0.15 M cacodylate buffer at pH 7.2) using a microwave with vacuum (BioWave Pelco). The larvae and adult tissue segments were incubated in the same fixative at 4 °C for one week and washed in 0.15 M cacodylate buffer with 3.5% mannitol.

Further EM processing was done according to an en bloc staining protocol, using a BioWave microwave [64]. The tissue was post-fixed in 2% osmium tetroxide (EMS, #19130) and 2 mM calcium chloride in 0.15 M sodium cacodylate (EMS, #12310), followed by a reducing step in 2.5% potassium hexacyanoferrate (Sigma, #14459-95-1). After impregnation with thiocarbohydrazide (1% in water, Sigma, #2231-57-4), the tissue was further contrasted by 2% aqueous osmium. 1% uranylacetate (EMS, #22400) was added overnight at 4 °C and for two hours at room temperature. Samples were dehydrated at increasing ethanol concentrations and embedded in LX112.

For SEM imaging, ultrathin sections at 100 nm thickness were generated on a Leica UC7 ultramicrotome and attached to carbon nanotube (CNT) tape strips (Science Services). SEM imaging for zebrafish spinal cord samples was performed on a Crossbeam 340 (Zeiss). Images were taken at 4 × 4 nm (larvae) and 10 × 10 nm (adult) pixel size, and analyzed using ImageJ.

### Confocal microscopy analysis

All quantifications of the confocal imaging experiments were done using IMARIS 9.9.0 (Bitplane) and FIJI 1.54p.

Myelinated area was quantified in Fiji from 6 × 1 tile scans of Tg(mbp:EGFP-CAAX) zebrafish spinal cords by measuring the total mbp:EGFP-CAAX^+^ pixel area within manually thresholded regions of interest from maximum intensity projection images.

Analysis of OPC and OL counts, total OL processes, number of myelin sheaths, myelin stubs, and ensheathed neuronal cell bodies, as well as the sheath lengths, was done manually on 3D volumes using IMARIS. For quantification of oligodendrocyte lineage cells, sox10:mRFP^+^ OPCs and newly-differentiated OLs that had their cell bodies in the dorsal hemi-spinal cord were included. Myelinating OLs were quantified from the dorsal spinal cord based on Tg(mbp:EGFP-CAAX) reporter expression. Cell body ensheathments were identified as almost-spherical, partial cup-shaped, or fully covered mbp:EGFP-CAAX^+^ membrane around a cell body that had no emerging processes, unlike the mbp:EGFP-CAAX^+^ OLs. For the sheath length analysis, myelin sheaths, defined as mbp:EGFP-CAAX^+^ cylindrical structures with a length >2 µm [16], of the dorsal spinal cord and commissural axons were included. Myelin stubs were defined as mbp:EGFP-CAAX^+^ (often blob-like) structures at the end of OL processes that did not match the criteria of a myelin sheath. Total processes per OL represent the sum of the number of OL processes connected to myelin sheaths, membrane stubs, and ensheathed cell bodies.

To quantify Caspr-EYFP localization, a binary readout of paranodal (i.e., Caspr localization at the lateral edges of myelin sheaths) versus internodal localization (i.e., uniform colocalization with sox10:mRFP reporter) was used to calculate percentage paranodal localization.

For the analyses of myelin growth and myelin sheath retraction dynamics, newly-myelinating OLs, sparse-labeled using myrf:EGFP-CAAX, were tracked over 13 hours. The time point at which the sparsely labeled OLs began forming axo-glial contacts and nascent myelin sheaths was identified as early-myelinating OL, and the number of early-myelinating OL processes was analyzed at this stage. At a time point 2 hours before the early-myelinating OL stage, the OPC processes were quantified. The maximum number of myelin sheaths formed per OL during the time-lapse was quantified by identifying myelin sheaths >2 µm in all the individual time frames using the 3D-view in IMARIS. The stabilized sheaths per OL were identified as the myelin sheaths >2 µm that remained at the end of the 13-hour time lapse. The percentage of retracted myelin sheaths is quantified as the percentage of sheaths lost at the end (13 hours) of the maximum number of myelin sheaths formed per OL during the time lapse.

For the OL cell death analysis, cells that began as a pre-myelinating OL and completed differentiation to form myelin sheaths were analyzed from time-lapse experiments. A binary readout (loss versus survival of the imaged OL) was obtained at the end of the 13-hour time lapse. OL that survived during the time-lapse session were included for myelin growth and retraction analysis.

### Electron microscopy analysis

For the EM analysis at 8 dpf, quantifications were done on whole spinal cords (one cross-section per fish) using FIJI. The total number of myelinated axons per µm^2^ was quantified as the number of myelinated axons in a cross-section of the spinal cord divided by the area of the spinal cord. Multi-myelin profiles and myelin outfoldings were quantified as a percentage of all the myelinated axons. Compact myelin was categorized as having densely-stained ensheathment around the axon, while the non-compact myelin was identified as lightly-stained membrane ensheathing the axon with distinguishable uncompacted cytoplasmic regions bordered by membranes.

The frequency distribution quantification of axonal diameters was done in FIJI. The freehand form was used to trace the axonal cross-sectional area, and the diameter was calculated from the measured area using the formula: diameter = 2 * √(area/*π*). About 95% of the myelinated axons were quantified in all groups, with some axons excluded due to fixation or tissue damage. The percentage of myelinated axons with an axonal diameter less than 1 µm was quantified as a percentage of the total number of myelinated axons traced. For the mean diameter of the 30 largest axons, the axonal cross-section area of around 50–70 axons, both myelinated and unmyelinated axons, was traced, and the quantifications include the 30 largest diameter measurements. The Mauthner axon diameter was quantified as the average of the two Mauthner axons per fish.

For the EM analysis at 2.5 months, quantifications were done on whole spinal cord sections (one cross-section per fish) and were done on QuPath. The myelinated axons were analyzed from four 900 µm^2^ fields of view (FOV), two each for the dorsal and ventral spinal cords of both hemispheres. The numbers of dorsal and ventral myelinated axons were added and averaged between the two hemispheres, and then divided by the measured area to be represented in the graph.

Representative images for the figures were prepared using FIJI, IMARIS (9.9.0), VAST, and Adobe Illustrator 2026. For the representative images of sox10:mRFP^+^ OPCs (Fig 3D), the 3D volume of the imaged spinal cord was cropped using IMARIS to exclude the myelin-dense dorsal spinal tract for the clear visualization of the OPCs.

### Statistical analysis

All statistical analyses were performed using GraphPad Prism 10.5.0. To test for normal distribution of the data, the D’Agostino–Pearson test or the Shapiro–Wilk test was used. For datasets with multiple experimental groups that were normally distributed, one-way ANOVA with Tukey’s multiple comparisons test was performed. For datasets with multiple experimental groups that did not pass the normal distribution test in GraphPad, the unpaired non-parametric Kruskal–Wallis test with Dunn’s multiple comparisons test was used. For datasets exhibiting substantial differences in standard deviations between the experimental groups, the Brown–Forsythe and Welch ANOVA with Dunnett’s T3 multiple comparison test was performed. The qPCR dataset was analyzed using the unpaired two-tailed non-parametric Mann–Whitney test. The statistical tests used for each comparison (and their significance level) are indicated in the figure legends. Statistical significance was considered when *p* < 0.05. *P* values are indicated as—*p < 0.05, **p < 0.01, ***p < 0.001, ****p < 0.0001. Graphs and data described in the results are presented as mean ± standard deviation (SD) or median with the interquartile range (IQR). “*n*” is used to indicate the number of cells and fish quantified per genotype, as referenced in the figure legends.

## Supporting information

S1 Fig Localization of internodal and paranodal adhesion molecules Mag, Cadm3, and Caspr in the zebrafish spinal cord. **(A)** Confocal image shows myelinating OL expressing the glial Mag fusion protein, labeled using olig1:mag-EGFP. Mag-EGFP (green) co-expressed with myelin reporter, sox10:mRFP (magenta), localizes to the leading edge of myelin, ensheathing the Mauthner axon. Arrowheads indicate the cytoplasm-rich areas of the spirally wrapping myelin sheaths. **(B)** Confocal image shows a myelinating OL in the dorsal spinal cord expressing glial Mag fusion protein (green) at 3 dpf together with a myelin reporter, sox10:mRFP (magenta). **(C)** Confocal image shows the accumulation of an axonal protein Cadm3 fusion protein (yellow), labeled using huC:cadm3-EGFP, at myelin internodes. Myelin sheaths are labeled using the reporter sox10:mRFP (magenta). **(D)** Selected frames from confocal time-lapse recording showing the re-distribution of Caspr-EYFP fusion protein (yellow) to paranodes (left: huC:Gal4;UAS:caspr-EYFP, right: Caspr-EYFP projected on myelin reporter, sox10:mRFP (magenta)). **(E)** Quantification of the percentage of Caspr-EYFP localization at the paranode of myelin sheaths at 2.5, 3, and 5 dpf. Graph represents mean values ± SD, analyzed by Brown-Forsythe and Welch ANOVA with Dunnett’s T3 multiple comparisons test. *p < 0.05, **p < 0.01, ****p < 0.0001. Scale bar is 10 µm for (A and B) and 5 µm for (C and D). Standard deviation projections are shown for (A–C), and maximum intensity projections are shown for (D). The data underlying this Figure can be found in S1 Data.
(TIF)

S2 Fig CRISPR-Cas9-mediated gene editing to create *cadm4* knockout. **(A)** Schematic of the *cadm4* gene, where exon 4 (highlighted in red) was chosen as the target exon for CRISPR-Cas9 gene editing. **(B)** Schematic of the AlphaFold predicted model, obtained from Ensembl, of Cadm4 protein domains in zebrafish. The arrowheads highlight the second extracellular Ig-like domain of the Cadm4 protein that is partly coded for by exon 4. **(C)** Gene sequence of exon 4 (wild-type allele) selected for CRISPR-Cas9-mediated gene editing. Green arrow indicates the cadm4 gRNA target sequence on the negative strand. Purple highlight indicates the PAM sequence. A frameshift mutation was induced by an 8-basepair (bp) deletion, resulting in a premature stop codon (indicated by red asterisk) in the *cadm4* knockout. The loss of the BciVI restriction enzyme recognition sequence confirmed the CRISPR indel mutation. The sequence displayed was obtained by Sanger sequencing. **(D)** Representative gel electrophoresis image of PCR, followed by restriction digestion-based validation of *cadm4* knockout in wild type and *CCM*^*−/−*^ tKO. Restriction digestion was performed using BfuI enzyme. The size of the molecular ladder is indicated. **(E)** Expected PCR and restriction digestion bands for PCR-based validation of KO. **(F)** qPCR quantification showing the relative *cadm4* mRNA expression in wild-type and *CCM*^*−/−*^ tKO zebrafish larvae. **(G)** qPCR quantification showing the relative mRNA expression of *cadm* gene family members in the *CCM*^*−/−*^ tKO. Data was collected from 10 fish per genotype. Graphs represent the median with the interquartile range, and statistical analysis was done using the unpaired two-tailed non-parametric Mann–Whitney test. *p < 0.05, **p < 0.01, ***p < 0.001, ****p < 0.0001. The data underlying this Figure can be found in S1 Data.
(TIF)

S3 Fig *CCM^-/-^* triple knockout shows exacerbated hypomyelination and abnormal myelin. **(A)** Quantification of the total number of myelinated axons per µm^2^ from the entire cross-section of 8 dpf zebrafish spinal cord in wild type, *cntn1b*^*−/−*^*mag*^*−/−*^ dKO, *cadm4*^*−/−*^*mag*^*−/−*^ dKO, and *CCM*^*−/−*^ tKO. **(B)** SEM images of non-compact and compact myelin sheaths at 8 dpf, identified for quantification in (C). Scale bars are 0.5 µm. **(C)** Quantification of the percentage of myelinated axons with compact myelin sheaths at 8 dpf. **(D)** SEM images of abnormal multi-myelin profiles and myelin outfoldings in zebrafish spinal cord cross-section at 8 dpf, identified for quantification in (E and F). Scale bars are 2 µm. **(E)** Quantification of the percentage of myelinated axons with abnormal multi-myelin profiles at 8 dpf. **(F)** Quantification of the percentage of myelin sheath outfoldings at 8 dpf. Graphs represent mean values ± SD, analyzed by one-way ANOVA with Tukey’s multiple comparisons test (A), and by Brown-Forsythe and Welch ANOVA with Dunnett’s T3 multiple comparisons test (C, E, F). *p < 0.05, **p < 0.01, ***p < 0.001, ****p < 0.0001. Data was collected from 5 to 6 fish (A, C, E, F) per genotype. The data underlying this Figure can be found in S1 Data.
(TIF)

S1 Video Caspr-EYFP re-distributes to pre-formed paranodes during myelin sheath growth in Tg(sox10:mRFP) fish at 3 dpf. Caspr-EYFP fusion protein (yellow) initially shows a uniform distribution across the myelin sheath (magenta), followed by redistribution exclusively to the paranode regions. Scalebar is 5 µm, time elapsed in hours. Time-lapse imaging was performed at 30 min intervals, and the video plays at 5 frames per second. (Associated with S1 Fig).
(AVI)

S2 Video Movie from time-lapse confocal imaging of myelin sheath retractions in wild type, labeled with myrf:EGFP-CAAX. OPCs progressing into myelinating OLs go through the early myelinating OL stage, where the processes make multiple axo-glial contacts and form nascent sheaths. Arrowheads indicate the retracting sheaths at 2.5–3.5 dpf. Scalebar is 10 µm, and time elapsed in hours. Time-lapse imaging was performed at 15 min intervals over 13 h and the video plays at 5 frames per second. (Associated with Fig 2).
(AVI)

S3 Video Movie from time-lapse confocal imaging of myelin sheath retractions in *cntn1b*^*−/−*^*mag*^*−/−*^ dKO, labeled with myrf:EGFP-CAAX at 2.5–3.5 dpf. Arrowheads indicate the retracting sheaths. Scalebar is 10 µm, and time elapsed in hours. Time-lapse imaging was performed at 15 min intervals over 13 h, and the video plays at 5 frames per second. (Associated with Fig 2).
(AVI)

S4 Video Movie from time-lapse confocal imaging of impaired myelin sheath retraction and cell body ensheathments in *cadm4*^*−/−*^*mag*^*−/−*^ dKO, labeled with myrf:EGFP-CAAX, at 2.5–3.5 dpf. The early myelinating OL forms multiple nascent sheaths that extend to form myelin sheaths. However, a large fraction of these sheaths is retracted away (indicated by arrowheads). Scalebar is 10 µm, and time elapsed in hours. Time-lapse imaging was performed at 15 min intervals over 13 h, and the video plays at 5 frames per second. (Associated with Fig 2).
(AVI)

S5 Video Movie from time-lapse confocal imaging of impaired myelin sheath retraction and cell body ensheathments in *CCM*^*−/−*^ tKO, labeled with myrf:EGFP-CAAX, at 2.5–3.5 dpf. In the *CCM*^*−/−*^ tKO, early myelinating OL forms multiple nascent sheaths, but only a small fraction of these grow and extend to form myelin sheaths. Additionally, many of the extended myelin sheaths are further retracted (indicated by arrowheads). Scalebar is 10 µm, and time elapsed in hours. Time-lapse imaging was performed at 15 min intervals over 13 h, and the video plays at 5 frames per second. (Associated with Fig 2).
(AVI)

S6 Video Movie from time-lapse confocal imaging of surviving OL, sparsely labeled using myrf:EGFP-CAAX, in wild type at 2.5–3.5 dpf. Scalebar is 10 µm, and time elapsed in hours. Time-lapse imaging was done at 15 min intervals over 13 h and the video plays at 5 frames per second. (Associated with Fig 3).
(AVI)

S7 Video Movie from time-lapse confocal imaging of surviving OL, sparsely labeled using myrf:EGFP-CAAX, in *cntn1b*^*−/−*^*mag*^*−/−*^ dKO at 2.5–3.5 dpf. Scalebar is 10 µm, and time elapsed in hours. Time-lapse imaging was performed at 15 min intervals over 13 h and the video plays at 5 frames per second. (Associated with Fig 3).
(AVI)

S8 Video Movie from time-lapse confocal imaging of OL undergoing cell death, sparsely labeled using myrf:EGFP-CAAX, in *cadm4*^*−/−*^*mag*^*−/−*^ dKO at 2.5–3.5 dpf. Early myelinating OL shows initial formation of myelin sheaths that are quickly retracted, followed by disintegration of the sheaths and cell death. Scalebar is 10 µm, and time elapsed in hours. Time-lapse imaging was performed at 15 min intervals over 13 h and the video plays at 5 frames per second. (Associated with Fig 3).
(AVI)

S9 Video Movie from time-lapse confocal imaging of OL undergoing cell death, sparsely labeled using myrf:EGFP-CAAX, in *CCM*^*−/−*^ tKO at 2.5–3.5 dpf. The fluorescence-labeled OL shows initial formation of myelin sheaths that quickly retract, followed by disintegration of the sheaths and cell death. Rare ectopic labeling of axons with myrf:EGFP-CAAX is present in the movie. Scalebar is 10 µm, and time elapsed in hours. Time-lapse imaging was performed at 15 min intervals over 13 h, and the video plays at 5 frames per second. (Associated with Fig 3).
(AVI)

S1 Raw Image Image of gel electrophoresis results for validation of CRISPR-Cas9-mediated indel of *cadm4* gene using Polymerase Chain Reaction - Restriction Fragment Length Polymorphism (PCR-RFLP). Gel electrophoresis image for *cadm4* genotyping, used to generate S2D Fig. The gel image was captured using Gel Doc XR+ Gel Documentation System (Bio-Rad), and was visualized using Image Lab 6.1 software (Bio-Rad). The figure was prepared using Adobe Illustrator 2026.
(TIF)

S1 Data Underlying data. (XLSX)

## Abbreviations

dpf: days post fertilization
MAG: Myelin-associated glycoprotein
Cadm4: Cell Adhesion Molecule 4
CNS: central nervous system
Cntn1: Contactin1
dKO: double knockout
FOV: fields of view
IQR: interquartile range
KO: knockout
OL: Oligodendrocytes
OPCs: oligodendrocyte precursor cells
PNS: peripheral nervous system
qPCR: quantitative PCR
RNP: ribonucleoprotein
ROI: regions of interest
SD: standard deviation
SEM: scanning electron microscopy
tKO: triple knockout

## References

[1] Stadelmann C, Timmler S, Barrantes-Freer A, Simons M. Myelin in the central nervous system: structure, function, and pathology. Physiol Rev. 2019;99(3):1381–431. doi: 10.1152/physrev.00031.2018 31066630

[2] Nave K-A, Werner HB. Myelination of the nervous system: mechanisms and functions. Annu Rev Cell Dev Biol. 2014;30:503–33. doi: 10.1146/annurev-cellbio-100913-013101 25288117

[3] Bechler ME, Byrne L, Ffrench-Constant C. CNS myelin sheath lengths are an intrinsic property of oligodendrocytes. Curr Biol. 2015;25(18):2411–6. doi: 10.1016/j.cub.2015.07.056 26320951 PMC4580335

[4] Lee S, Leach MK, Redmond SA, Chong SYC, Mellon SH, Tuck SJ, et al. A culture system to study oligodendrocyte myelination processes using engineered nanofibers. Nat Methods. 2012;9(9):917–22. doi: 10.1038/nmeth.2105 22796663 PMC3433633

[5] Mei F, Fancy SPJ, Shen Y-AA, Niu J, Zhao C, Presley B, et al. Micropillar arrays as a high-throughput screening platform for therapeutics in multiple sclerosis. Nat Med. 2014;20(8):954–60. doi: 10.1038/nm.3618 24997607 PMC4830134

[6] Hildebrand C, Remahl S, Persson H, Bjartmar C. Myelinated nerve fibres in the CNS. Prog Neurobiol. 1993;40(3):319–84. doi: 10.1016/0301-0082(93)90015-k 8441812

[7] Fields RD. A new mechanism of nervous system plasticity: activity-dependent myelination. Nat Rev Neurosci. 2015;16(12):756–67. doi: 10.1038/nrn4023 26585800 PMC6310485

[8] Almeida R, Lyons D. Oligodendrocyte development in the absence of their target axons in vivo. PLoS One. 2016;11(10):e0164432. doi: 10.1371/journal.pone.0164432 27716830 PMC5055324

[9] Bonetto G, Belin D, Káradóttir RT. Myelin: A gatekeeper of activity-dependent circuit plasticity?. Science. 2021;374(6569):eaba6905. doi: 10.1126/science.aba6905 34618550

[10] Monje M. Myelin plasticity and nervous system function. Annu Rev Neurosci. 2018;41:61–76. doi: 10.1146/annurev-neuro-080317-061853 29986163

[11] Almeida RG, Williamson JM, Madden ME, Early JJ, Voas MG, Talbot WS, et al. Myelination induces axonal hotspots of synaptic vesicle fusion that promote sheath growth. Curr Biol. 2021;31(17):3743-3754.e5. doi: 10.1016/j.cub.2021.06.036 34270947 PMC8445327

[12] Hines JH, Ravanelli AM, Schwindt R, Scott EK, Appel B. Neuronal activity biases axon selection for myelination in vivo. Nat Neurosci. 2015;18(5):683–9. doi: 10.1038/nn.3992 25849987 PMC4414883

[13] Mensch S, Baraban M, Almeida R, Czopka T, Ausborn J, El Manira A, et al. Synaptic vesicle release regulates myelin sheath number of individual oligodendrocytes in vivo. Nat Neurosci. 2015;18(5):628–30. doi: 10.1038/nn.3991 25849985 PMC4427868

[14] Wake H, Ortiz FC, Woo DH, Lee PR, Angulo MC, Fields RD. Nonsynaptic junctions on myelinating glia promote preferential myelination of electrically active axons. Nat Commun. 2015;6:7844. doi: 10.1038/ncomms8844 26238238 PMC4532789

[15] Almeida RG. The rules of attraction in central nervous system myelination. Front Cell Neurosci. 2018;12:367. doi: 10.3389/fncel.2018.00367 30374292 PMC6196289

[16] Almeida AR, Macklin WB. Early myelination involves the dynamic and repetitive ensheathment of axons which resolves through a low and consistent stabilization rate. Elife. 2023;12:e82111. doi: 10.7554/eLife.82111 37078701 PMC10198724

[17] Kirby BB, Takada N, Latimer AJ, Shin J, Carney TJ, Kelsh RN, et al. In vivo time-lapse imaging shows dynamic oligodendrocyte progenitor behavior during zebrafish development. Nat Neurosci. 2006;9(12):1506–11. doi: 10.1038/nn1803 17099706

[18] Schnädelbach O, Ozen I, Blaschuk OW, Meyer RL, Fawcett JW. N-cadherin is involved in axon-oligodendrocyte contact and myelination. Mol Cell Neurosci. 2001;17(6):1084–93. doi: 10.1006/mcne.2001.0961 11414796

[19] Laursen LS, Chan CW, ffrench-Constant C. An integrin-contactin complex regulates CNS myelination by differential Fyn phosphorylation. J Neurosci. 2009;29(29):9174–85. doi: 10.1523/JNEUROSCI.5942-08.2009 19625508 PMC4017644

[20] Linneberg C, Harboe M, Laursen LS. Axo-glia interaction preceding CNS myelination is regulated by bidirectional Eph-ephrin signaling. ASN Neuro. 2015;7(5):1759091415602859. doi: 10.1177/1759091415602859 26354550 PMC4568937

[21] Taveggia C, Zanazzi G, Petrylak A, Yano H, Rosenbluth J, Einheber S, et al. Neuregulin-1 type III determines the ensheathment fate of axons. Neuron. 2005;47(5):681–94. doi: 10.1016/j.neuron.2005.08.017 16129398 PMC2387056

[22] Michailov GV, Sereda MW, Brinkmann BG, Fischer TH, Haug B, Birchmeier C, et al. Axonal neuregulin-1 regulates myelin sheath thickness. Science (1979). 2004;304: 700–3. doi: 10.1126/SCIENCE.1095862 15044753

[23] Lundgaard I, Luzhynskaya A, Stockley JH, Wang Z, Evans KA, Swire M, et al. Neuregulin and BDNF induce a switch to NMDA receptor-dependent myelination by oligodendrocytes. PLoS Biol. 2013;11(12):e1001743. doi: 10.1371/journal.pbio.1001743 24391468 PMC3876980

[24] Charles P, Hernandez MP, Stankoff B, Aigrot MS, Colin C, Rougon G, et al. Negative regulation of central nervous system myelination by polysialylated-neural cell adhesion molecule. Proc Natl Acad Sci U S A. 2000;97(13):7585–90. doi: 10.1073/pnas.100076197 10840047 PMC16589

[25] Redmond SA, Mei F, Eshed-Eisenbach Y, Osso LA, Leshkowitz D, Shen YAA. Somatodendritic expression of JAM2 inhibits oligodendrocyte myelination. Neuron. 2016;91:824–36. doi: 10.1016/j.neuron.2016.07.021 27499083 PMC4990461

[26] Djannatian M, Timmler S, Arends M, Luckner M, Weil M-T, Alexopoulos I, et al. Two adhesive systems cooperatively regulate axon ensheathment and myelin growth in the CNS. Nat Commun. 2019;10(1):4794. doi: 10.1038/s41467-019-12789-z 31641127 PMC6805957

[27] Garcia MA, Zuchero JB. Anchors away: glia-neuron adhesion regulates myelin targeting and growth. Dev Cell. 2019;51(6):659–61. doi: 10.1016/j.devcel.2019.11.018 31951538

[28] Klingseisen A, Ristoiu A-M, Kegel L, Sherman DL, Rubio-Brotons M, Almeida RG, et al. Oligodendrocyte neurofascin independently regulates both myelin targeting and sheath growth in the CNS. Dev Cell. 2019;51(6):730-744.e6. doi: 10.1016/j.devcel.2019.10.016 31761670 PMC6912162

[29] Golan N, Kartvelishvily E, Spiegel I, Salomon D, Sabanay H, Rechav K, et al. Genetic deletion of Cadm4 results in myelin abnormalities resembling Charcot-Marie-Tooth neuropathy. J Neurosci. 2013;33(27):10950–61. doi: 10.1523/JNEUROSCI.0571-13.2013 23825401 PMC3718361

[30] Elazar N, Vainshtein A, Rechav K, Tsoory M, Eshed-Eisenbach Y, Peles E. Coordinated internodal and paranodal adhesion controls accurate myelination by oligodendrocytes. J Cell Biol. 2019;218(9):2887–95. doi: 10.1083/jcb.201906099 31451613 PMC6719437

[31] Elazar N, Vainshtein A, Golan N, Vijayaragavan B, Schaeren-Wiemers N, Eshed-Eisenbach Y, et al. Axoglial adhesion by Cadm4 regulates CNS myelination. Neuron. 2019;101(2):224-231.e5. doi: 10.1016/j.neuron.2018.11.032 30551998 PMC6371057

[32] Li C, Tropak MB, Gerlai R, Clapoff S, Abramow-Newerly W, Trapp B, et al. Myelination in the absence of myelin-associated glycoprotein. Nature. 1994;369(6483):747–50. doi: 10.1038/369747a0 7516497

[33] Montag D, Giese KP, Bartsch U, Martini R, Lang Y, Blüthmann H, et al. Mice deficient for the myelin-associated glycoprotein show subtle abnormalities in myelin. Neuron. 1994;13(1):229–46. doi: 10.1016/0896-6273(94)90472-3 7519026

[34] Maurel P, Einheber S, Galinska J, Thaker P, Lam I, Rubin MB, et al. Nectin-like proteins mediate axon Schwann cell interactions along the internode and are essential for myelination. J Cell Biol. 2007;178(5):861–74. doi: 10.1083/jcb.200705132 17724124 PMC2064549

[35] Sukhanov N, Vainshtein A, Eshed-Eisenbach Y, Peles E. Differential contribution of Cadm1-Cadm3 cell adhesion molecules to peripheral myelinated axons. J Neurosci. 2021;41(7):1393–400. doi: 10.1523/JNEUROSCI.2736-20.2020 33397712 PMC7896006

[36] Spiegel I, Adamsky K, Eshed Y, Milo R, Sabanay H, Sarig-Nadir O, et al. A central role for Necl4 (SynCAM4) in Schwann cell-axon interaction and myelination. Nat Neurosci. 2007;10(7):861–9. doi: 10.1038/nn1915 17558405 PMC2836764

[37] Bartsch U, Kirchhoff F, Schachner M. Immunohistological localization of the adhesion molecules L1, N-CAM, and MAG in the developing and adult optic nerve of mice. J Comp Neurol. 1989;284(3):451–62. doi: 10.1002/cne.902840310 2474006

[38] Eisenbach M, Kartvelishvily E, Eshed-Eisenbach Y, Watkins T, Sorensen A, Thomson C, et al. Differential clustering of Caspr by oligodendrocytes and Schwann cells. J Neurosci Res. 2009;87(15):3492–501. doi: 10.1002/jnr.22157 19565653

[39] Einheber S, Zanazzi G, Ching W, Scherer S, Milner TA, Peles E, et al. The axonal membrane protein Caspr, a homologue of neurexin IV, is a component of the septate-like paranodal junctions that assemble during myelination. J Cell Biol. 1997;139(6):1495–506. doi: 10.1083/jcb.139.6.1495 9396755 PMC2132621

[40] Pedraza L, Huang JK, Colman D. Disposition of axonal caspr with respect to glial cell membranes: implications for the process of myelination. J Neurosci Res. 2009;87(15):3480–91. doi: 10.1002/jnr.22004 19170162

[41] Almeida RG, Czopka T, Ffrench-Constant C, Lyons DA. Individual axons regulate the myelinating potential of single oligodendrocytes in vivo. Development. 2011;138(20):4443–50. doi: 10.1242/dev.071001 21880787 PMC3177314

[42] Chan JR, Jolicoeur C, Yamauchi J, Elliott J, Fawcett JP, Ng BK, et al. The polarity protein Par-3 directly interacts with p75NTR to regulate myelination. Science. 2006;314(5800):832–6. doi: 10.1126/science.1134069 17082460

[43] Meng X, Maurel P, Lam I, Heffernan C, Stiffler MA, McBeath G, et al. Necl-4/Cadm4 recruits Par-3 to the Schwann cell adaxonal membrane. Glia. 2019;67(5):884–95. doi: 10.1002/glia.23578 30585357 PMC7138615

[44] Heffernan C, Jain MR, Liu T, Kim H, Barretto K, Li H, et al. Nectin-like 4 complexes with choline transporter-like protein-1 and regulates schwann cell choline homeostasis and lipid biogenesis in vitro. J Biol Chem. 2017;292(11):4484–98. doi: 10.1074/jbc.M116.747816 28119456 PMC5377767

[45] Umemori H, Sato S, Yagi T, Aizawa S, Yamamoto T. Initial events of myelination involve Fyn tyrosine kinase signalling. Nature. 1994;367(6463):572–6. doi: 10.1038/367572a0 7509042

[46] Klein C, Kramer E-M, Cardine A-M, Schraven B, Brandt R, Trotter J. Process outgrowth of oligodendrocytes is promoted by interaction of fyn kinase with the cytoskeletal protein tau. J Neurosci. 2002;22(3):698–707. doi: 10.1523/JNEUROSCI.22-03-00698.2002 11826099 PMC6758498

[47] White R, Gonsior C, Krämer-Albers E-M, Stöhr N, Hüttelmaier S, Trotter J. Activation of oligodendroglial Fyn kinase enhances translation of mRNAs transported in hnRNP A2-dependent RNA granules. J Cell Biol. 2008;181(4):579–86. doi: 10.1083/jcb.200706164 18490510 PMC2386098

[48] Gonsior C, Binamé F, Frühbeis C, Bauer NM, Hoch-Kraft P, Luhmann HJ, et al. Oligodendroglial p130Cas is a target of Fyn kinase involved in process formation, cell migration and survival. PLoS One. 2014;9(2):e89423. doi: 10.1371/journal.pone.0089423 24586768 PMC3931761

[49] Barres BA, Hart IK, Coles HS, Burne JF, Voyvodic JT, Richardson WD, et al. Cell death and control of cell survival in the oligodendrocyte lineage. Cell. 1992;70(1):31–46. doi: 10.1016/0092-8674(92)90531-g 1623522

[50] Hughes EG, Orthmann-Murphy JL, Langseth AJ, Bergles DE. Myelin remodeling through experience-dependent oligodendrogenesis in the adult somatosensory cortex. Nat Neurosci. 2018;21(5):696–706. doi: 10.1038/s41593-018-0121-5 29556025 PMC5920726

[51] Kamen Y, Chapman TW, Piedra ET, Ciolkowski ME, Hill RA. Transient upregulation of procaspase-3 during oligodendrocyte fate decisions. J Neurosci. 2025;45(12):e2066242025. doi: 10.1523/JNEUROSCI.2066-24.2025 39837665 PMC11924999

[52] Sun LO, Mulinyawe SB, Collins HY, Ibrahim A, Li Q, Simon DJ, et al. Spatiotemporal control of CNS myelination by oligodendrocyte programmed cell death through the TFEB-PUMA axis. Cell. 2018;175(7):1811-1826.e21. doi: 10.1016/j.cell.2018.10.044 30503207 PMC6295215

[53] Djannatian M, Radha S, Weikert U, Safaiyan S, Wrede C, Deichsel C, et al. Myelination generates aberrant ultrastructure that is resolved by microglia. J Cell Biol. 2023;222(3):e202204010. doi: 10.1083/jcb.202204010 36637807 PMC9856851

[54] Krämer-Albers E-M, White R. From axon-glial signalling to myelination: the integrating role of oligodendroglial Fyn kinase. Cell Mol Life Sci. 2011;68(12):2003–12. doi: 10.1007/s00018-010-0616-z 21207100 PMC11114493

[55] Auer F, Vagionitis S, Czopka T. Evidence for myelin sheath remodeling in the CNS revealed by in vivo imaging. Curr Biol. 2018;28: 549–59.e3. doi: 10.1016/j.cub.2018.01.017 29429620

[56] Tomassy GS, Berger DR, Chen H-H, Kasthuri N, Hayworth KJ, Vercelli A, et al. Distinct profiles of myelin distribution along single axons of pyramidal neurons in the neocortex. Science. 2014;344(6181):319–24. doi: 10.1126/science.1249766 24744380 PMC4122120

[57] Mensch S, Baraban M, Almeida R, Czopka T, Ausborn J, El Manira A, et al. Synaptic vesicle release regulates myelin sheath number of individual oligodendrocytes in vivo. Nat Neurosci. 2015;18(5):628–30. doi: 10.1038/nn.3991 25849985 PMC4427868

[58] Marisca R, Hoche T, Agirre E, Hoodless LJ, Barkey W, Auer F, et al. Functionally distinct subgroups of oligodendrocyte precursor cells integrate neural activity and execute myelin formation. Nat Neurosci. 2020;23(3):363–74. doi: 10.1038/s41593-019-0581-2 32066987 PMC7292734

[59] Shen MW, Arbab M, Hsu JY, Worstell D, Culbertson SJ, Krabbe O, et al. Predictable and precise template-free CRISPR editing of pathogenic variants. Nature. 2018;563(7733):646–51. doi: 10.1038/s41586-018-0686-x 30405244 PMC6517069

[60] Labun K, Montague TG, Gagnon JA, Thyme SB, Valen E. CHOPCHOP v2: a web tool for the next generation of CRISPR genome engineering. Nucleic Acids Res. 2016;44(W1):W272-6. doi: 10.1093/nar/gkw398 27185894 PMC4987937

[61] Concordet J-P, Haeussler M. CRISPOR: intuitive guide selection for CRISPR/Cas9 genome editing experiments and screens. Nucleic Acids Res. 2018;46(W1):W242–5. doi: 10.1093/nar/gky354 29762716 PMC6030908

[62] Hruscha A, Schmid B. Generation of zebrafish models by CRISPR /Cas9 genome editing. Methods Mol Biol. 2015;1254:341–50. doi: 10.1007/978-1-4939-2152-2_24 25431076

[63] Kwan KM, Fujimoto E, Grabher C, Mangum BD, Hardy ME, Campbell DS, et al. The Tol2kit: a multisite gateway-based construction kit for Tol2 transposon transgenesis constructs. Dev Dyn. 2007;236(11):3088–99. doi: 10.1002/dvdy.21343 17937395

[64] Kislinger G, Niemann C, Rodriguez L, Jiang H, Fard MK, Snaidero N, et al. Neurons on tape: Automated Tape Collecting Ultramicrotomy-mediated volume EM for targeting neuropathology. Methods Cell Biol. 2023;177:125–70. doi: 10.1016/bs.mcb.2023.01.012 37451765

