# Supplementary figures and images for: Multiple adhesion molecules act together in oligodendrocyte-mediated axonal selection and myelin formation
Source: PLoS Biol. 2026 Jun 25;24(6):e3003854. doi: 10.1371/journal.pbio.3003854 (PMC13298782; doi:10.1371/journal.pbio.3003854)

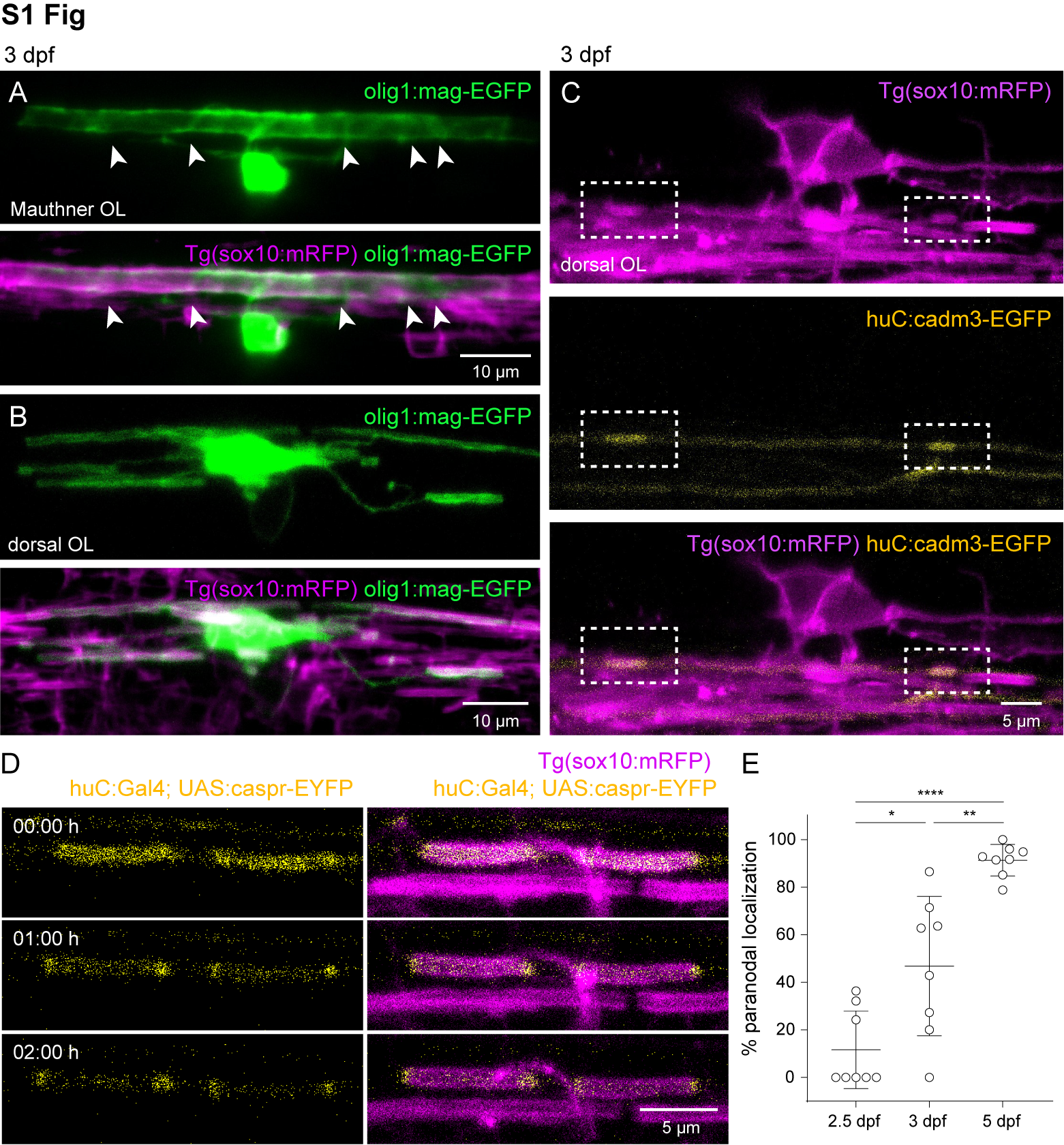

Supplement: S1 Fig — (A) Confocal image shows myelinating OL expressing the glial Mag fusion protein, labeled using olig1:mag-EGFP. Mag-EGFP (green) co-expressed with myelin reporter, sox10:mRFP (magenta), localizes to the leading edge of myelin, ensheathing the Mauthner axon. Arrowheads indicate the cytoplasm-rich areas of the spirally wrapping myelin sheaths. (B) Confocal image shows a myelinating OL in the dorsal spinal cord expressing glial Mag fusion protein (green) at 3 dpf together with a myelin reporter, sox10:mRFP (magenta). (C) Confocal image shows the accumulation of an axonal protein Cadm3 fusion protein (yellow), labeled using huC:cadm3-EGFP, at myelin internodes. Myelin sheaths are labeled using the reporter sox10:mRFP (magenta). (D) Selected frames from confocal time-lapse recording showing the re-distribution of Caspr-EYFP fusion protein (yellow) to paranodes (left: huC:Gal4;UAS:caspr-EYFP, right: Caspr-EYFP projected on myelin reporter, sox10:mRFP (magenta)). (E) Quantification of the percentage of Caspr-EYFP localization at the paranode of myelin sheaths at 2.5, 3, and 5 dpf. Graph represents mean values ± SD, analyzed by Brown-Forsythe and Welch ANOVA with Dunnett’s T3 multiple comparisons test. *p < 0.05, **p < 0.01, ****p < 0.0001. Scale bar is 10 µm for (A and B) and 5 µm for (C and D). Standard deviation projections are shown for (A–C), and maximum intensity projections are shown for (D). The data underlying this Figure can be found in S1 Data. (TIF) [file pbio.3003854.s001.tif]

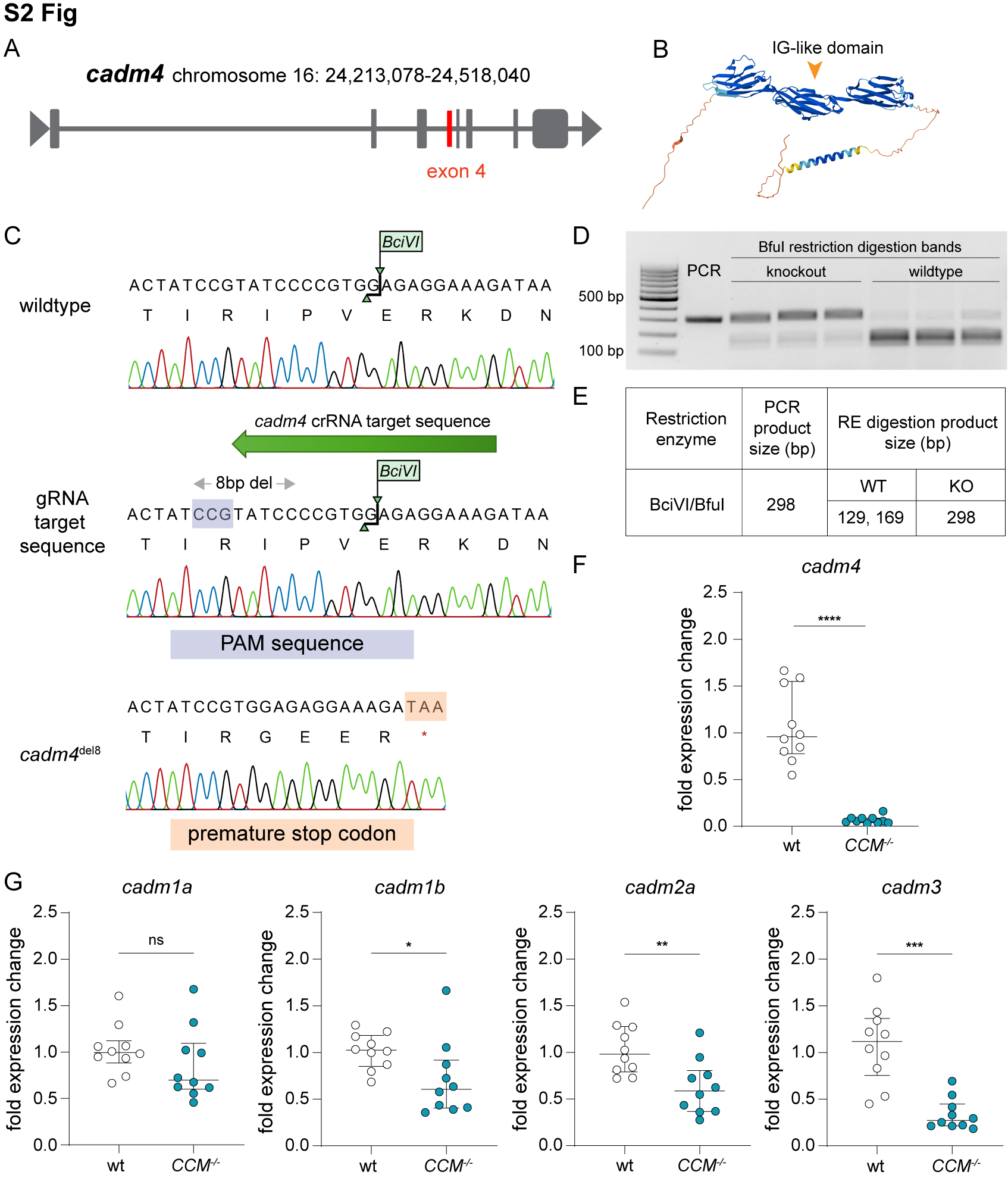

Supplement: S2 Fig — (A) Schematic of the cadm4 gene, where exon 4 (highlighted in red) was chosen as the target exon for CRISPR-Cas9 gene editing. (B) Schematic of the AlphaFold predicted model, obtained from Ensembl, of Cadm4 protein domains in zebrafish. The arrowheads highlight the second extracellular Ig-like domain of the Cadm4 protein that is partly coded for by exon 4. (C) Gene sequence of exon 4 (wild-type allele) selected for CRISPR-Cas9-mediated gene editing. Green arrow indicates the cadm4 gRNA target sequence on the negative strand. Purple highlight indicates the PAM sequence. A frameshift mutation was induced by an 8-basepair (bp) deletion, resulting in a premature stop codon (indicated by red asterisk) in the cadm4 knockout. The loss of the BciVI restriction enzyme recognition sequence confirmed the CRISPR indel mutation. The sequence displayed was obtained by Sanger sequencing. (D) Representative gel electrophoresis image of PCR, followed by restriction digestion-based validation of cadm4 knockout in wild type and CCM−/− tKO. Restriction digestion was performed using BfuI enzyme. The size of the molecular ladder is indicated. (E) Expected PCR and restriction digestion bands for PCR-based validation of KO. (F) qPCR quantification showing the relative cadm4 mRNA expression in wild-type and CCM−/− tKO zebrafish larvae. (G) qPCR quantification showing the relative mRNA expression of cadm gene family members in the CCM−/− tKO. Data was collected from 10 fish per genotype. Graphs represent the median with the interquartile range, and statistical analysis was done using the unpaired two-tailed non-parametric Mann–Whitney test. *p < 0.05, **p < 0.01, ***p < 0.001, ****p < 0.0001. The data underlying this Figure can be found in S1 Data. (TIF) [file pbio.3003854.s002.tif]

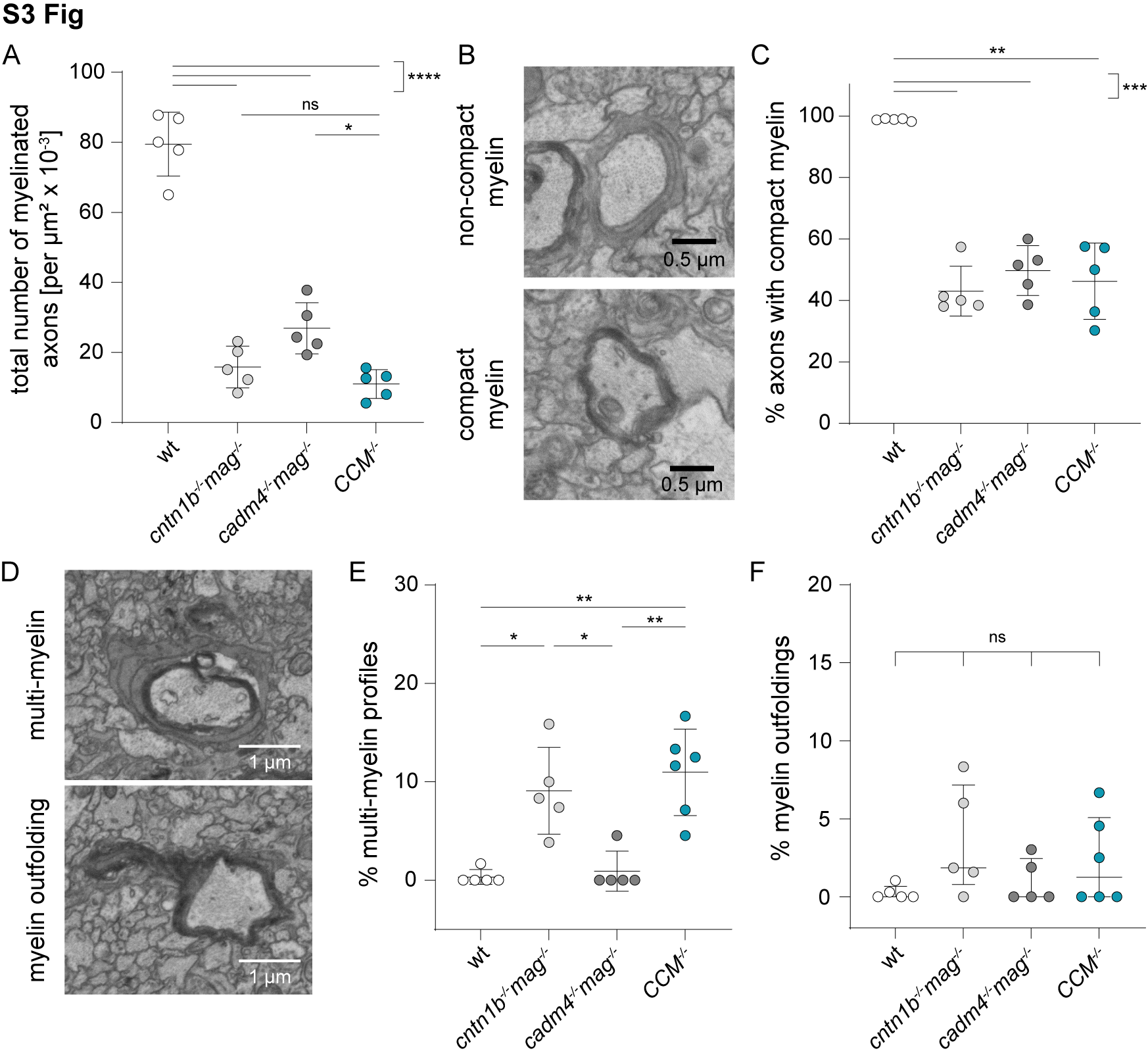

Supplement: S3 Fig — (A) Quantification of the total number of myelinated axons per µm2 from the entire cross-section of 8 dpf zebrafish spinal cord in wild type, cntn1b−/−mag−/− dKO, cadm4−/−mag−/− dKO, and CCM−/− tKO. (B) SEM images of non-compact and compact myelin sheaths at 8 dpf, identified for quantification in (C). Scale bars are 0.5 µm. (C) Quantification of the percentage of myelinated axons with compact myelin sheaths at 8 dpf. (D) SEM images of abnormal multi-myelin profiles and myelin outfoldings in zebrafish spinal cord cross-section at 8 dpf, identified for quantification in (E and F). Scale bars are 2 µm. (E) Quantification of the percentage of myelinated axons with abnormal multi-myelin profiles at 8 dpf. (F) Quantification of the percentage of myelin sheath outfoldings at 8 dpf. Graphs represent mean values ± SD, analyzed by one-way ANOVA with Tukey’s multiple comparisons test (A), and by Brown-Forsythe and Welch ANOVA with Dunnett’s T3 multiple comparisons test (C, E, F). *p < 0.05, **p < 0.01, ***p < 0.001, ****p < 0.0001. Data was collected from 5 to 6 fish (A, C, E, F) per genotype. The data underlying this Figure can be found in S1 Data. (TIF) [file pbio.3003854.s003.tif]

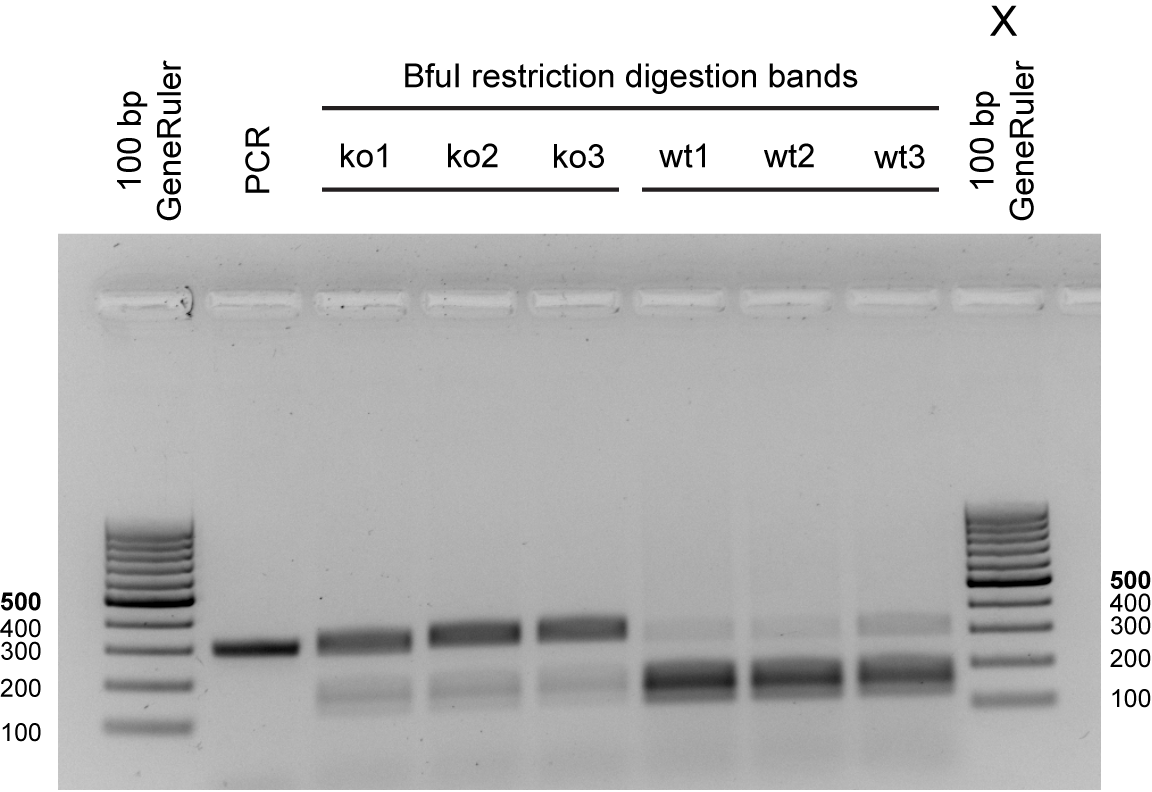

Supplement: S1 Raw Image — Gel electrophoresis image for cadm4 genotyping, used to generate S2D Fig. The gel image was captured using Gel Doc XR+ Gel Documentation System (Bio-Rad), and was visualized using Image Lab 6.1 software (Bio-Rad). The figure was prepared using Adobe Illustrator 2026. (TIF) [file pbio.3003854.s013.tif]
