# Supplementary material for: Multiple adhesion molecules act together in oligodendrocyte-mediated axonal selection and myelin formation
Source: PLoS Biol. 2026 Jun 25;24(6):e3003854. doi: 10.1371/journal.pbio.3003854 (PMC13298782; doi:10.1371/journal.pbio.3003854)
Supplement: Attachment — Submitted filename: PBIOLOGY-D-26-00129R1_Response-to-Reviewers_v2.pdf [file pbio.3003854.s015.pdf]

We thank all three reviewers for their time and effort in carefully evaluating our manuscript and for their insightful comments that have improved the quality of the manuscript.

Reviewer #1: In this manuscript by the Simons group, Radha et al. report that the orchestration of multiple neuronal and oligodendroglial cell adhesion molecules are required for myelin sheath formation and the subsequent survival of the differentiating oligodendrocyte.

To make their discoveries the authors use in vivo live cell imaging and electron microscopy on combined zebrafish mutants of the adhesion molecules cadm4 (oligodendrocyte encoded), MAG (oligodendrocyte encoded), and cntn1b (neuron encoded). The triple mutants show severe dysmyelination in the spinal cord (Fig 1). Analysis of individual oligodendrocyte morphologies revealed that cells failed to establish and extend nascent myelin sheaths (Fig 2). Moreover, through timelapse analysis of differentiating oligodendrocytes during the axon selection process the authors reveal increased cell death, presumably through failure of successful integration (Fig 3). Lastly, they present through electron microscopic analysis that the myelination failure preferentially affected smaller diameter axons (and that the triple mutants exhibit a general increase in axon diameter) (Fig 4).

Overall, this is a highly interesting and very strong manuscript. Interesting, because to date no single molecule has been identified as required to fate axons for myelination in the central nervous system. This is in strong contrast to the peripheral nervous system where it is established since over 20 years that this choice is critically regulated by levels neuregulin expression. Here, the authors provide compelling evidence there may simply be no single molecule to regulate the same process in the CNS. Instead, it appears to be the combination of multiple and somewhat redundant adhesion mechanisms that are required. Hence, the authors provide insights into a long-standing question in the field.
Experimental evidence for their findings is strong because the phenotypes presented are compelling and the analysis is thorough throughout. The authors combine multiple CRISPR mutants and compare all phenotypes seen in the triple mutants to combinations of double mutants. Furthermore, the supplementary figures and videos provide interesting supporting information on the localisation of the different adhesion molecules and the cellular phenotypes that mutant oligodendrocytes exhibit. I find the observation that differentiating oligodendrocytes die when they cannot establish proper adhesion to target axons particularly striking! While a link between myelination and oligodendrocyte survival is of course established as the authors rightly state, this is at least to my knowledge the first time where this has been documented so precisely.

For these reasons, I don't think that this manuscript requires any major changes before acceptance. I only have a few thoughts that may be worth including in the discussion:
- the authors discuss that failure of sheath stabilisation might lead to lysosomal overload, stress and subsequent death of the oligodendrocyte. Would it be worth discussing alternative scenarios as to why oligodendrocytes die when they fail to establish proper ensheathment of target axons?

Author response: We thank the reviewer for this valuable comment. We have now extended the discussion (see page 14).

"We have previously shown that retracting sheath fragments and the resulting debris accumulates within lysosomes of the OL soma [53]. Consequently, the failure to stabilize sheaths *CCM*$^{-/-}$ tKO may lead to lysosomal overload with lipid-rich myelin debris, inducing cellular stress and promoting cell death. Alternatively, OL survival may depend on axon-derived trophic signals [8], such that loss or reduction of these cues could contribute to the observed cell death, for example by disrupting downstream signaling pathways that are critical during the transition to the myelinating state [54]."

- The authors report that their triple and double mutants exhibit an increase in axon diameter even though there is less myelination. Overall, axon diameter and myelination are somewhat linked / positively correlated, so this observation is interesting because it is counterintuitive. It may be worth including this discussion point.

Author response: We would like to emphasize that the observed increase in axon diameter was primarily found in large-caliber axons of the ventral spinal cord, which are characteristic of reticulospinal projections. As our analysis was not based on 3D electron microscopy volumes, we are unable to determine whether this increase reflects focal enlargements or a uniform change along the length of the axon. We have now expanded the discussion of this limitation (see page 14).

"Notably, a subset of large-diameter axons belonging to reticulospinal neurons exhibited increased caliber in both *cntn1b*$^{-/-}$*mag*$^{-/-}$ dKOs and *CCM*$^{-/-}$ tKO. We therefore hypothesize that the observed increase in axon diameter reflects cytoskeletal remodeling, possibly arising from reduced cortical tension due to diminished oligodendrocyte-axon adhesion. Because this phenotype affected only a small subset of axons, axonal enlargement alone cannot account for the loss of small-diameter myelination in *CCM*$^{-/-}$ tKOs."

- page 5, bottom third: "Notably, myelin content in CCM-/- tKOs showed only a minimal increase by 8 dpf". Do the authors mean increase or decrease?

Author response: We have now rephrased. We wanted to point out that myelin content in the *CCM*$^{-/-}$ tKO only slightly increases between 4 and 8 dpf, and thus represents a persistent phenotype throughout developmental myelination.

Reviewer #2: This manuscript by Radha et al. presents new data that builds upon previous work which showed that axo-glial adhesion is required beginning during the earliest stages of myelin formation for myelin sheath-axon engagement, sheath stabilization, and subsequently for sheath elongation. Cadm4, Cntn1b, and Mag have been previously shown to regulate CNS myelination, and in this manuscript, they show for the first time that combined loss of all three of these molecules has a more profound effect on myelin sheath formation than in single or double knockouts, indicating redundant functions. The authors generated a new cadm4 loss-of-function zebrafish mutant, which by itself only had a minimal effect on myelination, but loss of cadm4 had a stronger effect when combined as a double or triple knockout with cntn1b and mag mutations. Total myelin area is reduced most significantly in tKOs, and total OL processes (defined as processes that connect to myelin sheaths, stubs, or

myelin wrapped cell bodies) are most significantly reduced tKOs. The processes they do have generate more myelin stubs (that reflect failure to initiate myelination) and retractions, and this leads to increased OL cell death and subsequently fewer OLs present. When myelin sheaths in tKOs are present they have reduced compaction and increased double myelin and outfoldings. The authors conclude that combined loss of Cadm4, Cntn1b, and Mag are together required to establish healthy, stable myelin sheaths and prevent ectopic myelination of cell bodies. The work is strong and the paper compelling and will be of broad interest to the glial and myelin fields.

Concerns:

1. One of the authors' important points is that loss of all three adhesion molecules resulted in less targeting of myelin to axons <1um, and while the data supports that conclusion, the tKOs also have a striking increase in the diameter of axons. The authors should provide some explanation about why axon diameters are increased in cntn1b-/-;mag-/- dKO and in tKO. Does the myelinated axon frequency by diameter curves just reflect that there are more larger diameter axons (Fig 4C)?

Author response: Thank you for the important question. As also suggested by reviewer 3, we tried to quantify unmyelinated axons, but this analysis was not possible because unmyelinated small-diameter axons could not be reliably distinguished from processes of other cells. However, we would like to point out that the enlarged diameters are mainly seen in large-diameter axons in the ventral spinal cord, so it seems to selectively affect one axon subpopulation. Therefore, axon enlargement itself does not explain the loss of small-diameter myelination.

We currently don't know whether the increased axon diameters that we see in the ventral spinal cord represent rather focal enlargements or are uniform along the length of the axon. We speculate that the loss of adhesion molecules in *cntn1b*$^{-/-}$*;mag*$^{-/-}$ dKO and *CCM*$^{-/-}$ tKO leads to a disorganization of cytoskeletal components, such as actin, neurofilaments, and microtubules, as well as a loss of cortical tension. This might both cause axon enlargement. We have now extended the discussion on this issue (see page 14).

2. The data shows that cntn1b-/-;mag-/- dKO have 23% OL death from 2.5-3.5dpf but not significantly fewer Mbp+ OLs at 4dpf, yet no compensatory increase in Sox10+ OLs is shown. This should be commented on.

Author response: Thank you for raising this important point. At present, we can only speculate on the underlying mechanisms. One possibility is that the loss of OPCs is rapidly compensated by increased migration and differentiation of neighboring OPCs within the zebrafish spinal cord. In the *cntn1b*$^{-/-}$*; mag*$^{-/-}$ double knockout, the observed 23% reduction in early-myelinating oligodendrocytes between 2.5–3.5 dpf occurs during a period of active OPC migration, differentiation, and maturation. Consequently, this loss may be transient and rapidly compensated, allowing cell numbers to return to homeostatic levels by 4 dpf (Fig. 3E). A more direct assessment of these dynamic replacement processes would require quantification of oligodendrocyte lineage stages, such as OPCs, pre-myelinating oligodendrocytes, and myelinating oligodendrocytes, along defined differentiation and

maturation trajectories. However, this analysis was not feasible in the current study due to the limited availability of stage-specific reporter lines.

3. The amount of cell death of cadm4-/-; mag-/- OLs should be reported since there is a ~50% reduction in OL number in these dKOs, or some comment about what happened to them.

Author response: Thank you for the important point. We have included an additional time-lapse experiment and analysis of cell death in the *cadm4*$^{-/-}$*mag*$^{-/-}$ dKO in Fig. 3A-B. Interestingly, 56% of early-myelinating *cadm4*$^{-/-}$*mag*$^{-/-}$ dKO OLs underwent cell death, comparable to that in the *CCM*$^{-/-}$ tKO. This result is in line with the observed reduction in both *cadm4*$^{-/-}$*mag*$^{-/-}$ dKO *and CCM*$^{-/-}$ tKO at 4 dpf, as shown in Fig. 3C-E. Both mutants exhibit an exceptionally high number of sheath retractions (Fig. 3L), and we speculate that this may lead to a lysosomal overload, thereby promoting cell death. Alternatively, early-myelinating OLs may require supportive signals from the axons, and the increased retractions may prevent the OL from receiving enough of these signals to ensure its survival.

4. The authors state that there are fewer initial OL process extensions/branches per OL in the tKOs, which they have defined as processes that connect to myelin sheaths, stubs, or myelin wrapped cell bodies. It does appear that the tKO OLs actually dynamically extend fewer processes in the video during the earliest stages when processes are looking for axons to target. If there are fewer process extensions then maybe there is just less chance for axon contact, in addition to reduced sheath stabilization. I think they only counted "processes" as ones attached to sheaths, stubs or cell bodies. Perhaps they should include quantification of dynamic process extensions per OL.

Author response: Thank you for the excellent suggestion. We agree that this is crucial information to dissect the mechanisms of myelination failure in the absence of the different adhesion proteins. We therefore further analysed our previous time-lapse imaging of individual myelinating OLs, sparsely labelled using myrf:EGFP-CAAX, and added new acquisitions for the *cadm4*$^{-/-}$*mag*$^{-/-}$ dKO. Different stages across the OPC differentiation and myelination were identified for the analysis. Myelinating OLs forming sheaths were identified at the end of the 13-hour time-lapse. Early-myelinating OLs were identified at the time point where they formed multiple processes and nascent myelin sheaths appeared. OPCs were characterized with a morphology containing multiple long processes, approximately 2 hours prior to the early-myelinating OL stage (Fig. 2G).

We found that the OPCs in the double and triple knockouts extended processes comparable to the wildtype (Fig. 2H). Further, at the beginning of myelination, marked by the appearance of nascent sheaths, the number of processes extended by the early-myelinating OLs appears to be the same in the knockouts and wildtype (Fig. 2I). Strikingly, myelinating OLs in *cadm4*$^{-/-}$*mag*$^{-/-}$ dKO made maximum myelin sheaths comparable to wildtype, but the majority of these sheaths were retracted, so that the final number of processes at 4 dpf was comparable to the ones observed in *CCM*$^{-/-}$ tKOs (Fig 2B). In contrast, myelinating OLs in the *CCM*$^{-/-}$ tKO displayed an impaired initiation of myelin sheaths in combination with high sheath retraction rates, leading to a severe deficit of stable sheaths formed by individual OLs (Fig. 2J-L).

Additionally, the representative images for the time-lapse imaging of *CCM*$^{-/-}$ tKO OL in the preliminary manuscript (Fig 2A, Video 4) were modified to accurately match the developmental stage of the OPCs to the myelinating OLs transition, since the previous data did not record the sparsely-labelled OL from the OPC stage.

Minor concerns:

1. The sox10:mRFP images in Fig. 3G (4 dpf) don't show myelin sheaths, which should be visible at this stage with this transgene and are visible with the same transgene as early as 3 dpf in Fig. S1B, and the cells visible in the panels appear to be mostly OPCs. Is the time-point shown in the figure legend accurate?

Author response: We thank the reviewer for pointing out this issue. We confirm that the image in Fig. 3G (Fig. 3D in the revised manuscript) corresponds to the time point 4dpf, where Sox10^{+} OPCs and early-myelinating OLs were quantified. At this time point, the dorsal axonal tracts were already densely myelinated, which made it difficult to distinguish individual Sox10^{+} OPCs. Therefore, we cropped the lateral parts of the spinal cord, where these tracts reside, using IMARIS. We included a comment in the legends and methods.

Below is the uncropped image of the same samples at 4dpf that includes the dorsal myelinated axonal tract.

Fig 3D

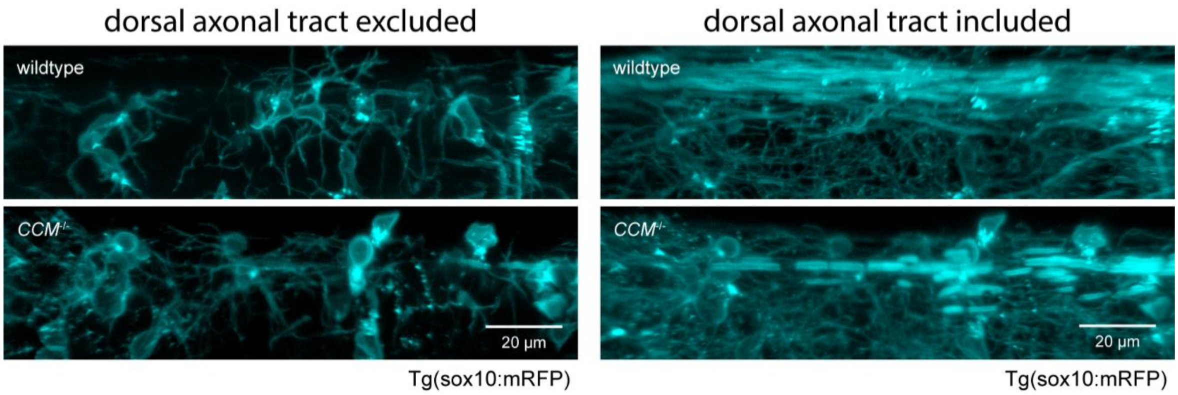

2. The authors reference the review of the Klingseisen et al. "Oligodendrocyte Neurofascin Independently Regulates Both Myelin Targeting and Sheath Growth in the CNS" paper but don't reference the paper itself.

Author response: We have incorporated the reference to the original research article in the Introduction.

Reviewer #3: The study of Radha et al reports three major oligodendrocyte (OLG) cell adhesion molecules (CAMs) i.e., Mag, Cadm4 and Cntn1, cooperate to initiate and stabilize OLG myelination. The authors generated double and triple (MCC) knockouts (KOs) in zebrafish and assessed myelination by live imaging an OLG reporter line and by EM. The resultant KO phenotypes are striking, particularly in the MCC KO, which exhibit reduced numbers of OLG processes, "stubby" myelin, and an increase in OLG fragmentation/death. The study extends two prior reports [1, 2] that characterized these CAMs by single or double

KOs in mice and in zebrafish and provided robust evidence for defects of axon and myelin organization e.g., myelin segments that grew over other myelinated fibers, over nodes of Ranvier, and around neuronal cell bodies. Similar findings, including myelination of neuronal somata, are reported here, although the overall characterization is more limited. The major novelty of this report is the triple KO and the characterization of the more severe phenotype that results. Other major new findings of this study are not explicitly stated, but appear to include characterizing the development of the myelin mutant phenotype, documentation of OLG loss, and evidence of an increase in axon diameters. Together these new data provide additional support for the notion that combinatorial axo-glial adhesion systems of different domains ensure proper myelination. I have some concerns the authors should address as noted below.

One question is whether defects in the CAM KOs are entirely a failure of axon-glial adhesion as proposed here or may reflect other mechanisms, including OLG-autonomous effects. The major data marshalled in support of disrupted axo-glial adhesion here is the reduction of OLG processes and short myelin stubs. It is unclear if this reduction in processes is a failure of stable attachment to axons as suggested or may reflect changes in intracellular signaling, organization of the OLG cytoskeleton, proteostasis etc. that impacts OLG process formation and/or sheath elongation. As the authors note, OLGs myelinate synthetic fibers and do so without forming short stubby myelin segments despite a lack of ligands for OLG CAMs indicating lack of specific axo-glial adhesion by itself does not phenocopy their mutants.

Author response: We thank the reviewer for this valuable comment. We have now extended the discussion on this issue as suggested by the reviewer (see pages 13 and 14).

It would be useful to know if the reduction of myelin and OLG processes results from a failure to maintain processes that grow out initially and are later retracted (consistent with a failure of adhesion) or from a failure to form processes at all. Consistent with the latter possibility, some OLGs in the CCM KOs have reduced numbers of processes initially (see for ex. the OLG in video 4), with an abnormal OLG soma morphology. Quantification of the overall numbers of OLG processes per OLG at these two different time points, independent of whether they have myelin stubs or not, would be useful to better understand these defects.

Author response: We thank the reviewer for the excellent suggestion. To differentiate the failure to initiate myelin formation vs. sheath retractions, further analyzed our previous time-lapse imaging of individual myelinating OLs, sparsely labelled using myrf:EGFP-CAAX, and added new acquisitions for the *cadm4*$^{-/-}$*mag*$^{-/-}$ dKO. Different stages across the OPC differentiation and myelination were identified for the analysis. Myelinating OLs forming sheaths were identified at the end of the 13-hour time-lapse. Early-myelinating OLs were identified at the time point where they formed multiple processes and nascent myelin sheaths appeared. OPCs were characterized with a morphology containing multiple long processes, approximately 2 hours prior to the early-myelinating OL stage (Fig. 2G).

We found that the OPCs in the double and triple knockouts extended processes comparable to the wildtype (Fig. 2H). Further, at the beginning of myelination, marked by the appearance of nascent sheaths, the number of processes extended by the early-myelinating OLs appears to be the same in the knockouts and wildtype (Fig. 2I). Strikingly, myelinating OLs in *cadm4*$^{-/-}$

mag$^{-/-}$ dKO made maximum myelin sheaths comparable to wildtype, but the majority of these sheaths were retracted, so that the final number of processes at 4dpf was comparable to the ones observed in *CCM*$^{-/-}$ tKOs (Fig 2B). In contrast, myelinating OLs in the *CCM*$^{-/-}$ tKO displayed an impaired initiation of myelin sheaths in combination with high sheath retraction rates, leading to a severe deficit of stable sheaths formed by individual OLs (Fig. 2J-L).

Additionally, the representative images for the time-lapse imaging of *CCM*$^{-/-}$ tKO OL in the preliminary manuscript (Fig 2A, Video 4) were modified to accurately match the developmental stage of the OPCs to the myelinating OLs transition, since the previous data did not record the sparsely-labelled OL from the OPC stage.

In the Fig. 2 legend, the authors state that "data was collected from 15-17 individual oligodendrocytes from 9-15 fish per genotype". It is unclear if they quantified 15-17 OLGs per fish per genotype or a grand total of 15-17 per genotype (roughly 1 or 2 OLGs per fish). If the latter, this seems very modest and potentially subject to selection bias. The authors should clarify.

Author response: We quantified a total number of 15-17 oligodendrocytes per genotype, which were sampled from 9-11 (not 9-15) fish per genotype. This analysis required the identification of all processes emerging from an individual myelinating OL. In the wildtype and the *cadm4*$^{-/-}$ and *mag*$^{-/-}$ KOs, where myelination in the dorsal spinal cord was abundant, this analysis was challenging. Hence, we quantified only individual OLs where all the processes can be verifiably traced to the corresponding OL cell body, thus limiting our sample selection. We have now increased the total number of OLs as much as possible, to a total number of 16 (wt) and 20-22 (other genotypes).

Axons that are myelinated are skewed to those with larger diameters. This is plausibly interpreted as evidence that smaller axons are less likely to be myelinated in the KOs. However this interpretation is potentially complicated as the tKOs increase the diameters of myelinated axons as shown in Fig. 4E, F, with the overall diameter of myelinated axons shifted to the right (4C). Whether this reflects a failure to myelinate smaller axons or an increase in axon diameters is therefore unclear. To distinguish between these possibilities, it may be useful to determine if there is a greater increase in the percentages of small axons (< 1 µm) vs. larger axons that are unmyelinated in the mutants.

Author response: We thank the reviewer for the excellent suggestion. We tried to quantify unmyelinated axons, but this analysis was not possible, because unmyelinated small-diameter axons could not be reliably distinguished from processes of other cells. We therefore present the data as myelination/area. However, we would like to point out that the enlarged diameters are mainly seen in large-diameter axons in the ventral spinal cord, so it seems to selectively affect one axon subpopulation. Therefore, axon enlargement itself does not explain the loss of small-diameter myelination.

Are the myelin "stubs" and reduction in OLG processes primarily a zebrafish phenomenon? The study of Elazar et al [2] OLG processes aberrantly extend to contact other myelinated axons rather than retract or form small stubs in my reading. The authors should comment.

References:
1. Djannatian, M., et al., Two adhesive systems cooperatively regulate axon ensheathment and myelin growth in the CNS. Nat Commun, 2019. 10(1): p. 4794.
2. Elazar, N., et al., Coordinated internodal and paranodal adhesion controls accurate myelination by oligodendrocytes. J Cell Biol, 2019. 218(9): p. 2887-2895.

Author response: The study of Elazar et al. does not contain any data on individual oligodendrocytes, from which parameters like OL branching or myelin "stubs" could be drawn. In our own previous study (Djannatian et al., 2019), in which we introduced the phenotype of *cntn1b*$^{-/-}$*mag*$^{-/-}$, we directly compared myelination in mouse and zebrafish. The parallel use of these two model systems showed us that myelin phenotypes largely overlapped across species (pronounced hypomyelination, double myelin) and allowed us to identify underlying mechanisms by intravital imaging in zebrafish (revealing reduced sheath growth) and 3D electron microscopy (revealing two parallel phenotypes with similar appearance on 2D cross-sections: 1) sheath overgrowth, and 2) miswrapping phenotype). We speculate that the multi-myelin phenotypes in Cadm4$^{-/-}$/Mag$^{-/-}$ and Cadm4$^{-/-}$/Caspr$^{-/-}$ mice in the study of Elazar et al. represent a combination of sheath overgrowth and miswrapping as well.

We see two discrepancies between the Cadm4$^{-/-}$/Mag$^{-/-}$, as described by Elazar et al., and *cadm4*$^{-/-}$*mag*$^{-/-}$ zebrafish in the present study. First, *cadm4*$^{-/-}$*mag*$^{-/-}$ zebrafish at 8 dpf exhibited a reduction of myelin content of >50% in the spinal cord, whereas only a tendency towards less myelin was observed in the corpus callosum of 3-month-old *Cadm4*$^{-/-}$*/Mag*$^{-/-}$ mice. Second, while Elazar et al. et al. reported multi-myelin profiles in 8% of the myelinated *Cadm4*$^{-/-}$*/Mag*$^{-/-}$ axons, we only observed a comparable number of these in 1 out of 5 fish (Fig. S3E). These discrepancies could be attributed to different regions and/or developmental time points, or different roles of Cadm4 across species. We included an appropriate comment in the manuscript (pages 14 and 15).
