# Supplementary material for: Multiple adhesion molecules act together in oligodendrocyte-mediated axonal selection and myelin formation
Source: PLoS Biol. 2026 Jun 25;24(6):e3003854. doi: 10.1371/journal.pbio.3003854 (PMC13298782; doi:10.1371/journal.pbio.3003854)
Supplement: Peer review history

# Editor report: Decision Letter 0

**DOI:** 10.1371/journal.pbio.3003854.r001

**Authors:** Taylor Hart, PhD

22 Jan 2026

Dear Dr Simons,

Thank you for submitting your manuscript entitled "Multiple adhesion molecules act together in oligodendrocyte-mediated axonal selection and myelin formation" for consideration as a Short Report by PLOS Biology.

Your manuscript has now been evaluated by the PLOS Biology editorial staff, as well as by an academic editor with relevant expertise, and I am writing to let you know that we would like to send your submission out for external peer review.

However, before we can send your manuscript to reviewers, we need you to complete your submission by providing the metadata that is required for full assessment. To this end, please login to Editorial Manager where you will find the paper in the 'Submissions Needing Revisions' folder on your homepage. Please click 'Revise Submission' from the Action Links and complete all additional questions in the submission questionnaire.

Once your full submission is complete, your paper will undergo a series of checks in preparation for peer review. After your manuscript has passed the checks it will be sent out for review. To provide the metadata for your submission, please Login to Editorial Manager (https://www.editorialmanager.com/pbiology) within two working days, i.e. by Jan 24 2026 11:59PM.

If your manuscript has been previously peer-reviewed at another journal, PLOS Biology is willing to work with those reviews in order to avoid re-starting the process. Submission of the previous reviews is entirely optional and our ability to use them effectively will depend on the willingness of the previous journal to confirm the content of the reports and share the reviewer identities. Please note that we reserve the right to invite additional reviewers if we consider that additional/independent reviewers are needed, although we aim to avoid this as far as possible. In our experience, working with previous reviews does save time.

If you would like us to consider previous reviewer reports, please edit your cover letter to let us know and include the name of the journal where the work was previously considered and the manuscript ID it was given. In addition, please upload a response to the reviews as a 'Prior Peer Review' file type, which should include the reports in full and a point-by-point reply detailing how you have or plan to address the reviewers' concerns.

During the process of completing your manuscript submission, you will be invited to opt-in to posting your pre-review manuscript as a bioRxiv preprint. Visit http://journals.plos.org/plosbiology/s/preprints for full details. If you consent to posting your current manuscript as a preprint, please upload a single Preprint PDF.

Feel free to email us at plosbiology@plos.org if you have any queries relating to your submission.

Kind regards,

Taylor

Taylor Hart, PhD,

Associate Editor

PLOS Biology

thart@plos.org

# Peer review: Decision Letter 1

**DOI:** 10.1371/journal.pbio.3003854.r002

**Authors:** Taylor Hart, PhD, Taylor Hart, PhD

4 Mar 2026

Dear Dr Simons,

Thank you for your patience while your manuscript "Multiple adhesion molecules act together in oligodendrocyte-mediated axonal selection and myelin formation" went through peer-review at PLOS Biology. Your manuscript has now been evaluated by the PLOS Biology editors, an Academic Editor with relevant expertise, and by several independent reviewers. In light of the reviews, found at the end of this email, we would like to invite you to perform a revision that we do not think will take you very long.

As you will see, the reviewers commented on the strength of the reported phenotype and expressed interest in the findings. The reviewers' major points were related to the increase in axon diameters and the dynamics of oligodendrocyte process extensions. They also brought up areas where more data should be shown, additional points should be discussed, or other clarifications were needed. In your revision, you should provide a thorough response to the reviewers' points, including through textual revisions and further analysis of existing data.

After we receive your revision, we will then assess your revised manuscript and your response to the reviewers' comments with our Academic Editor aiming to avoid further rounds of peer-review, although we might need to consult with the reviewers, depending on the nature of the revisions.

In addition to these revisions, you may need to complete some formatting changes, which you will receive in a follow up email. A member of our team will be in touch with a set of requests shortly. If you do not receive a separate email within a few days, please assume that checks have been completed, and no additional changes are required.

We expect to receive your revised manuscript within 1 month. Please email us (plosbiology@plos.org) if you have any questions or concerns, or would like to request an extension.

At this stage, your manuscript remains formally under active consideration at our journal; please notify us by email if you do not intend to submit a revision so that we withdraw the manuscript.

**IMPORTANT - SUBMITTING YOUR REVISION**

Your revisions should address the specific points made by each reviewer. Please submit the following files along with your revised manuscript:

1. A 'Response to Reviewers' file - this should detail your responses to the editorial requests, present a point-by-point response to all of the reviewers' comments, and indicate the changes made to the manuscript.

*NOTE: In your point-by-point response to the reviewers, please provide the full context of each review. Do not selectively quote paragraphs or sentences to reply to. The entire set of reviewer comments should be present in full and each specific point should be responded to individually.

You should also cite any additional relevant literature that has been published since the original submission and mention any additional citations in your response.

2. In addition to a clean copy of the manuscript, please also upload a 'track-changes' version of your manuscript that specifies the edits made. This should be uploaded as a "Revised Article with Changes Highlighted " file type.

*Resubmission Checklist*

When you are ready to resubmit your revised manuscript, please refer to this resubmission checklist: https://plos.io/Biology_Checklist

To submit a revised version of your manuscript, please go to https://www.editorialmanager.com/pbiology/ and log in as an Author. Click the link labelled 'Submissions Needing Revision' where you will find your submission record.

Please make sure to read the following important policies and guidelines while preparing your revision:

*Published Peer Review*

Please note while forming your response, if your article is accepted, you may have the opportunity to make the peer review history publicly available. The record will include editor decision letters (with reviews) and your responses to reviewer comments. If eligible, we will contact you to opt in or out. Please see here for more details:

https://blogs.plos.org/plos/2019/05/plos-journals-now-open-for-published-peer-review/

*PLOS Data Policy*

Please note that as a condition of publication PLOS' data policy (http://journals.plos.org/plosbiology/s/data-availability) requires that you make available all data used to draw the conclusions arrived at in your manuscript. If you have not already done so, you must include any data used in your manuscript either in appropriate repositories, within the body of the manuscript, or as supporting information (N.B. this includes any numerical values that were used to generate graphs, histograms etc.). For an example see here: http://www.plosbiology.org/article/info%3Adoi%2F10.1371%2Fjournal.pbio.1001908#s5

*Blot and Gel Data Policy*

We require the original, uncropped and minimally adjusted images supporting all blot and gel results reported in an article's figures or Supporting Information files. We will require these files before a manuscript can be accepted so please prepare them now, if you have not already uploaded them. Please carefully read our guidelines for how to prepare and upload this data: https://journals.plos.org/plosbiology/s/figures#loc-blot-and-gel-reporting-requirements

*Protocols deposition*

To enhance the reproducibility of your results, we recommend that if applicable you deposit your laboratory protocols in protocols.io, where a protocol can be assigned its own identifier (DOI) such that it can be cited independently in the future. Additionally, PLOS ONE offers an option for publishing peer-reviewed Lab Protocol articles, which describe protocols hosted on protocols.io. Read more information on sharing protocols at https://plos.org/protocols?utm_medium=editorial-email&utm_source=authorletters&utm_campaign=protocols

Thank you again for your submission to our journal. We hope that our editorial process has been constructive thus far, and we welcome your feedback at any time. Please don't hesitate to contact us if you have any questions or comments.

Sincerely,

Taylor

Taylor Hart, PhD,

Associate Editor

PLOS Biology

thart@plos.org

----------------------------------------------------------------

REVIEWS:

Reviewer #1 [Tim Czopka]: In this manuscript by the Simons group, Radha et al. report that the orchestration of multiple neuronal and oligodendroglial cell adhesion molecules are required for myelin sheath formation and the subsequent survival of the differentiating oligodendrocyte.

To make their discoveries the authors use in vivo live cell imaging and electron microscopy on combined zebrafish mutants of the adhesion molecules cadm4 (oligodendrocyte encoded), MAG (oligodendrocyte encoded) and cntn1b (neuron encoded). The triple mutants show severe dysmyelination in the spinal cord (Fig 1). Analysis of individual oligodendrocyte morphologies revealed that cells failed to establish and extend nascent myelin sheaths (Fig 2). Moreover, through timelapse analysis of differentiating oligodendrocytes during the axon selection process the authors reveal increased cell death, presumably through failure of successful integration (Fig 3). Lastly, they present through electron microscopic analysis that the myelination failure preferentially affected smaller diameter axons (and that the triple mutants exhibit a general increase in axon diameter) (Fig 4).

Overall, this is a highly interesting and very strong manuscript. Interesting, because to date no single molecule has been identified as required to fate axons for myelination in the central nervous system. This is in strong contrast to the peripheral nervous system where it is established since over 20 years that this choice is critically regulated by levels neuregulin expression. Here, the authors provide compelling evidence there may simply be no single molecule to regulate the same process in the CNS. Instead, it appears to be the combination of multiple and somewhat redundant adhesion mechanisms that are required. Hence, the authors provide insights into a long-standing question in the field.

Experimental evidence for their findings is strong because the phenotypes presented are compelling and the analysis is thorough throughout. The authors combine multiple CRISPR mutants and compare all phenotypes seen in the triple mutants to combinations of double mutants. Furthermore, the supplementary figures and videos provide interesting supporting information on the localisation of the different adhesion molecules and the cellular phenotypes that mutant oligodendrocytes exhibit. I find the observation that differentiating oligodendrocytes die when they cannot establish proper adhesion to target axons particularly striking! While a link between myelination and oligodendrocyte survival is of course established as the authors rightly state, this is at least to my knowledge the first time where this has been documented so precisely.

For these reasons, I don't think that this manuscript requires any major changes before acceptance. I only have a few thoughts that may be worth including in the discussion:

- the authors discuss that failure of sheath stabilisation might lead to lysosomal overload, stress and subsequent death of the oligodendrocyte. Would it be worth discussing alternative scenarios as to why oligodendrocytes die when they fail to establish proper ensheathment of target axons?

- The authors report that their triple and double mutants exhibit an increase in axon diameter even though there is less myelination. Overall, axon diameter and myelination are somewhat linked / positively correlated, so this observation is interesting because it is counterintuitive. It may be worth including this discussion point.

- page 5, bottom third: "Notably, myelin content in CCM-/- tKOs showed only a minimal increase by 8 dpf". Do the authors mean increase or decrease?

Tim Czopka

Reviewer #2: This manuscript by Radha et al. presents new data that builds upon previous work which showed that axo-glial adhesion is required beginning during the earliest stages of myelin formation for myelin sheath-axon engagement, sheath stabilization, and subsequently for sheath elongation. Cadm4, Cntn1b, and Mag have been previously shown to regulate CNS myelination, and in this manuscript they show for the first time that combined loss of all three of these molecules has a more profound effect on myelin sheath formation than in single or double knockouts, indicating redundant functions. The authors generated a new cadm4 loss-of-function zebrafish mutant, which by itself only had a minimal effect on myelination, but loss of cadm4 had a stronger effect when combined as a double or triple knockout with cntn1b and mag mutations. Total myelin area is reduced most significantly in tKOs, and total OL processes (defined as processes that connect to myelin sheaths, stubs, or myelin wrapped cell bodies) are most significantly reduced tKOs. The processes they do have generate more myelin stubs (that reflect failure to initiate myelination) and retractions, and this leads to increased OL cell death and subsequently fewer OLs present. When myelin sheaths in tKOs are present they have reduced compaction and increased double myelin and outfoldings. The authors conclude that combined loss of Cadm4, Cntn1b, and Mag are together required to establish healthy, stable myelin sheaths and prevent ectopic myelination of cell bodies. The work is strong and the paper compelling and will be of broad interest to the glial and myelin fields.

Concerns:

1. One of the authors' important points is that loss of all three adhesion molecules resulted in less targeting of myelin to axons <1um, and while the data supports that conclusion, the tKOs also have a striking increase in the diameter of axons. The authors should provide some explanation about why axon diameters are increased in cntn1b-/-;mag-/- dKO and in tKO. Does the myelinated axon frequency by diameter curves just reflect that there are more larger diameter axons (Fig 4C)?

2. The data shows that cntn1b-/-;mag-/- dKO have 23% OL death from 2.5-3.5dpf but not significantly fewer Mbp+ OLs at 4dpf, yet no compensatory increase in Sox10+ OLs is shown. This should be commented on.

3. The amount of cell death of cadm4-/-; mag-/- OLs should be reported since there is a ~50% reduction in OL number in these dKOs, or some comment about what happened to them.

4. The authors state that there are fewer initial OL process extensions/branches per OL in the tKOs, which they have defined as processes that connect to myelin sheaths, stubs, or myelin wrapped cell bodies. It does appear that the tKO OLs actually dynamically extend fewer processes in the video during the earliest stages when processes are looking for axons to target. If there are fewer process extensions then maybe there is just less chance for axon contact, in addition to reduced sheath stabilization. I think they only counted "processes" as ones attached to sheaths, stubs or cell bodies. Perhaps they should include quantification of dynamic process extensions per OL.

Minor concerns:

1. The sox10:mRFP images in Fig. 3G (4 dpf) don't show myelin sheaths, which should be visible at this stage with this transgene and are visible with the same transgene as early as 3 dpf in Fig. S1B, and the cells visible in the panels appear to be mostly OPCs. Is the time-point shown in the figure legend accurate?

2. The authors reference the review of the Klingseisen et al. "Oligodendrocyte Neurofascin Independently Regulates Both Myelin Targeting and Sheath Growth in the CNS" paper but don't reference the paper itself.

Reviewer #3: The study of Radha et al reports three major oligodendrocyte (OLG) cell adhesion molecules (CAMs) i.e., Mag, Cadm4 and Cntn1, cooperate to initiate and stabilize OLG myelination. The authors generated double and triple (MCC) knockouts (KOs) in zebrafish and assessed myelination by live imaging an OLG reporter line and by EM. The resultant KO phenotypes are striking, particularly in the MCC KO, which exhibit reduced numbers of OLG processes, "stubby" myelin, and an increase in OLG fragmentation/death. The study extends two prior reports [1, 2] that characterized these CAMs by single or double KOs in mice and in zebrafish and provided robust evidence for defects of axon and myelin organization e.g., myelin segments that grew over other myelinated fibers, over nodes of Ranvier, and around neuronal cell bodies. Similar findings, including myelination of neuronal somata, are reported here, although the overall characterization is more limited. The major novelty of this report is the triple KO and the characterization of the more severe phenotype that results. Other major new findings of this study are not explicitly stated, but appear to include characterizing the development of the myelin mutant phenotype, documentation of OLG loss, and evidence of an increase in axon diameters. Together these new data provide additional support for the notion that combinatorial axo-glial adhesion systems of different domains ensure proper myelination. I have some concerns the authors should address as noted below.

One question is whether defects in the CAM KOs are entirely a failure of axon-glial adhesion as proposed here or may reflect other mechanisms, including OLG-autonomous effects. The major data marshalled in support of disrupted axo-glial adhesion here is the reduction of OLG processes and short myelin stubs. It is unclear if this reduction in processes is a failure of stable attachment to axons as suggested or may reflect changes in intracellular signaling, organization of the OLG cytoskeleton, proteostasis etc. that impacts OLG process formation and/or sheath elongation. As the authors note, OLGs myelinate synthetic fibers and do so without forming short stubby myelin segments despite a lack of ligands for OLG CAMs indicating lack of specific axo-glial adhesion by itself does not phenocopy their mutants.

It would be useful to know if the reduction of myelin and OLG processes results from a failure to maintain processes that grow out initially and are later retracted (consistent with a failure of adhesion) or from a failure to form processes at all. Consistent with the latter possibility, some OLGs in the CCM KOs have reduced numbers of processes initially (see for ex. the OLG in video 4), with an abnormal OLG soma morphology. Quantification of the overall numbers of OLG processes per OLG at these two different time points, independent of whether they have myelin stubs or not, would be useful to better understand these defects.

In the Fig. 2 legend, the authors state that "data was collected from 15-17 individual oligodendrocytes from 9-15 fish per genotype". It is unclear if they quantified 15-17 OLGs per fish per genotype or a grand total of 15-17 per genotype (roughly 1 or 2 OLGs per fish). If the latter, this seems very modest and potentially subject to selection bias. The authors should clarify.

Axons that are myelinated are skewed to those with larger diameters. This is plausibly interpreted as evidence that smaller axons are less likely to be myelinated in the KOs. However this interpretation is potentially complicated as the tKOs increase the diameters of myelinated axons as shown in Fig. 4E, F, with the overall diameter of myelinated axons shifted to the right (4C). Whether this reflects a failure to myelinate smaller axons or an increase in axon diameters is therefore unclear. To distinguish between these possibilities, it may be useful to determine if there is a greater increase in the percentages of small axons (< 1 µm) vs. larger axons that are unmyelinated in the mutants.

Are the myelin "stubs" and reduction in OLG processes primarily a zebrafish phenomenon? The study of Elazar et al [2] OLG processes aberrantly extend to contact other myelinated axons rather than retract or form small stubs in my reading. The authors should comment

References:

1. Djannatian, M., et al., Two adhesive systems cooperatively regulate axon ensheathment and myelin growth in the CNS. Nat Commun, 2019. 10(1): p. 4794.

2. Elazar, N., et al., Coordinated internodal and paranodal adhesion controls accurate myelination by oligodendrocytes. J Cell Biol, 2019. 218(9): p. 2887-2895.

# Author comment: Author response to Decision Letter 2

**DOI:** 10.1371/journal.pbio.3003854.r003

5 May 2026

Attachment Submitted filename: PBIOLOGY-D-26-00129R1_Response-to-Reviewers_v2.pdf

# Editor report: Decision Letter 2

**DOI:** 10.1371/journal.pbio.3003854.r004

**Authors:** Taylor Hart, PhD, Taylor Hart, PhD, Taylor Hart, PhD

14 May 2026

Dear Dr Simons,

Thank you for your patience while we considered your revised manuscript "Multiple adhesion molecules act together in oligodendrocyte-mediated axonal selection and myelin formation" for publication as a Short Report at PLOS Biology. This revised version of your manuscript has been evaluated by the PLOS Biology editors and the Academic Editor.

Based on our Academic Editor's assessment of your revision, we are likely to accept this manuscript for publication. Please also make sure to address the following data and other policy-related requests.

IMPORTANT: Please ensure that your next revision addresses the following points:

-----------------

**Financial disclosure statement:

Please add links to the funding agencies in the Financial Disclosure statement in the manuscript details.

**Competing interests:

Please modify the Competing Interests statement in the submission form to acknowledge that Dr Simons is on the PLOS Biology editorial board. You can use the following wording:

“MS is a member of PLOS Biology’s Editorial Board. The other authors declare that no competing interests exist."

**Ethics:

Please include the specific national or international regulations/guidelines to which your animal care and use protocol adhered.

**Data:

Thank you for providing the underlying data. Please cite the location of the data clearly in all relevant main and supplementary Figure legends, e.g. “The data underlying this Figure can be found in S1 Data”.

**Supplement:

As the supplementary figures appear to each be uploaded twice (once as .tif files and once as .pdf), please only include the image files.

-----------------

As you address these items, please take this last chance to review your reference list to ensure that it is complete and correct. If you have cited papers that have been retracted, please include the rationale for doing so in the manuscript text, or remove these references and replace them with relevant current references. Any changes to the reference list should be mentioned in the cover letter that accompanies your revised manuscript.

In addition to these revisions, you may need to complete some formatting changes, which you will receive in a follow up email. A member of our team will be in touch with a set of requests shortly. If you do not receive a separate email within a few days, please assume that checks have been completed, and no additional changes are required.

We expect to receive your revised manuscript within two weeks.

To submit your revision, please go to https://www.editorialmanager.com/pbiology/ and log in as an Author. Click the link labelled 'Submissions Needing Revision' to find your submission record. Your revised submission must include the following:

- a cover letter that should detail your responses to any editorial requests, if applicable, and whether changes have been made to the reference list

- a Response to Reviewers file that provides a detailed response to the reviewers' comments (if applicable, if not applicable please do not delete your existing 'Response to Reviewers' file.)

- a track-changes file indicating any changes that you have made to the manuscript.

NOTE: If Supporting Information files are included with your article, note that these are not copyedited and will be published as they are submitted. Please ensure that these files are legible and of high quality (at least 300 dpi) in an easily accessible file format. For this reason, please be aware that any references listed in an SI file will not be indexed. For more information, see our Supporting Information guidelines:

https://journals.plos.org/plosbiology/s/supporting-information

*Published Peer Review History*

Please note that you may have the opportunity to make the peer review history publicly available. The record will include editor decision letters (with reviews) and your responses to reviewer comments. If eligible, we will contact you to opt in or out. Please see here for more details:

https://plos.org/published-peer-review-history/

*Press*

Should you, your institution's press office or the journal office choose to press release your paper, please ensure you have opted out of Early Article Posting on the submission form. We ask that you notify us as soon as possible if you or your institution is planning to press release the article.

*Protocols deposition*

To enhance the reproducibility of your results, we recommend that if applicable you deposit your laboratory protocols in protocols.io, where a protocol can be assigned its own identifier (DOI) such that it can be cited independently in the future. Additionally, PLOS ONE offers an option for publishing peer-reviewed Lab Protocol articles, which describe protocols hosted on protocols.io. Read more information on sharing protocols at https://plos.org/protocols?utm_medium=editorial-email&utm_source=authorletters&utm_campaign=protocols

Please do not hesitate to contact me should you have any questions.

Sincerely,

Taylor

Taylor Hart, PhD,

Associate Editor

thart@plos.org

PLOS Biology

# Author comment: Author response to Decision Letter 3

**DOI:** 10.1371/journal.pbio.3003854.r005

22 May 2026

Attachment Submitted filename: PBIOLOGY-D-26-00129R1_Response-to-Reviewers_v2_auresp_3.pdf

# Editor report: Decision Letter 3

**DOI:** 10.1371/journal.pbio.3003854.r006

**Authors:** Taylor Hart, PhD, Taylor Hart, PhD, Taylor Hart, PhD, Taylor Hart, PhD

29 May 2026

Dear Dr Simons,

Thank you for the submission of your revised Short Report "Multiple adhesion molecules act together in oligodendrocyte-mediated axonal selection and myelin formation" for publication in PLOS Biology. On behalf of my colleagues and the Academic Editor, Ben Emery, I am pleased to say that we can in principle accept your manuscript for publication, provided you address any remaining formatting and reporting issues. These will be detailed in an email you should receive within 2-3 business days from our colleagues in the journal operations team; no action is required from you until then. Please note that we will not be able to formally accept your manuscript and schedule it for publication until you have completed any requested changes.

Please take a minute to log into Editorial Manager at http://www.editorialmanager.com/pbiology/, click the "Update My Information" link at the top of the page, and update your user information to ensure an efficient production process.

PRESS

We frequently collaborate with press offices. If your institution or institutions have a press office, please notify them about your upcoming paper at this point, to enable them to help maximise its impact. If the press office is planning to promote your findings, we would be grateful if they could coordinate with biologypress@plos.org. If you have previously opted in to the early version process, we ask that you notify us immediately of any press plans so that we may opt out on your behalf.

We also ask that you take this opportunity to read our Embargo Policy regarding the discussion, promotion and media coverage of work that is yet to be published by PLOS. As your manuscript is not yet published, it is bound by the conditions of our Embargo Policy. Please be aware that this policy is in place both to ensure that any press coverage of your article is fully substantiated and to provide a direct link between such coverage and the published work. For full details of our Embargo Policy, please visit http://www.plos.org/about/media-inquiries/embargo-policy/.

Thank you again for choosing PLOS Biology for publication and supporting Open Access publishing. We look forward to publishing your study.

Sincerely,

Taylor

Taylor Hart, PhD,

Associate Editor

PLOS Biology

thart@plos.org
